# Unraveling the Role of Ensheathing Cells and Perineural Fibroblasts in Olfactory Neurogenesis

**DOI:** 10.1002/glia.70076

**Published:** 2025-08-07

**Authors:** Katja Senf, Sandor Nietzsche, Martin Westermann, Eva M. Neuhaus

**Affiliations:** ^1^ Pharmacology and Toxicology Jena University Hospital, Friedrich Schiller University Jena Jena Germany; ^2^ Centre for Electron Microscopy Jena University Hospital, Friedrich Schiller University Jena Jena Germany

**Keywords:** lipid metabolism, olfactory ensheathing cell, olfactory epithelium, regeneration, satellite glia cells

## Abstract

During development and following injury‐induced neurogenesis, olfactory ensheathing cells (OECs) envelope the axon bundles of sensory neurons and support their growth to the glomerular destinations in the olfactory bulb. Transplantation of OECs to various neuronal injury locations showed a reparative impact; however, there was huge variability. By combining mRNA sequencing with bioinformatics analysis and immunohistochemistry, we characterized the cellular and molecular biological properties of OECs of the lamina propria and their response to neuronal injury. We found that OECs do not express NGFR (p75) under steady state conditions, questioning the common approach of isolating OECs with NGFR antibodies. While OECs express a peculiar combination of markers of different types of glial cells, they are strikingly similar to satellite glia cells of the dorsal root ganglion; for example, they showed marked upregulation of genes involved in lipid metabolism during neuronal regeneration. Similar to satellite glia cells and unlike Schwann cells, adult OECs did not proliferate in response to injury. Like endothelial cells at the blood–brain barrier and unlike other glia types, OECs showed extensive connections via the tight junction protein Claudin 5. Furthermore, OECs lack water channels, which probably explains why they sustain a stable environment after olfactory epithelium ablation. Regulation of the extracellular osmolarity seems to involve Aquaporin 1 in perineural fibroblasts together with high levels of KCNJ10, Na^+^K^+^ATPase, and gap junctions in OECs. Optimizing the clinical uses of these unique glia cells is probably made easier by this thorough characterization of marker gene expression in steady state and during neurogenesis.

## Main Points

- DEOs are similar to satellite glial cells.
- DECs react to neuronal injury by promoting lipid metabolism, but do not proliferate.
- ECs reduce gap junctions upon neuronal injury.
- Perineural fibroblasts also contribute to the injury response.

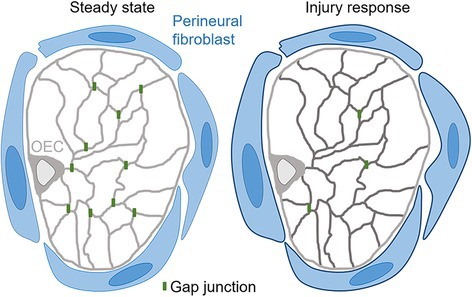

## 1 Introduction

Traumatic brain injury or spinal cord injury in humans and other mammals is usually devastating due to the limited repair ability of the central nervous system (CNS), while amphibians and teleost fish have an intrinsic ability to regenerate their axons (Becker et al. 1997; Sofroniew 2018). Peripheral nerves, however, can regenerate to some extent. Especially, the olfactory system has the ability to regenerate functional sensory neurons throughout life, supported by glia cells called olfactory ensheathing cells (OECs). OECs are present in the lamina propria of the olfactory mucosa and in the nerve layer of the olfactory bulb. Olfactory bulb OECs are localized at the transitional zone between the peripheral and CNS and seem to exhibit properties of both CNS and peripheral glia (Beiersdorfer et al. 2020).

The key peripheral nervous system glia are Schwann cells that populate most peripheral nerves, OECs, which are present in the lamina propria of the olfactory system and in the nerve layer of the olfactory bulb, satellite glia cells in peripheral ganglia, and enteric glia in the enteric nervous system surrounding the intestinal tract. Similar to other peripheral glia cells (Jacob et al. 2014; Jessen and Mirsky 2005; Serbedzija et al. 1991), OECs are derived from the neural crest (Barraud et al. 2010; Forni et al. 2011). OECs are responsible for facilitating axonal growth from the epithelium to the olfactory bulb by ensheathing large bundles of axons of olfactory neurons and supporting axon extension from the periphery into the appropriate glomeruli in the olfactory bulb. OECs express adhesion molecules that can promote axonal growth, such as NCAM, laminin, and tenascin (Kafitz and Greer 1998), and communicate via glutamatergic and purinergic pathways (Rieger et al. 2007). Motility of axons is regulated by migration of OECs and stimulation of OEC motility enhances axon extension in vitro (Windus et al. 2011). After removal of the olfactory bulb, OECs migrate to the injury site and rapidly phagocytose the axonal debris (Nazareth et al. 2015; Su et al. 2013). The phagocytic clearance of cell debris in the olfactory nerve appears to be performed solely by OECs, not by macrophages (Su et al. 2013), while in other peripheral nerves, Schwann cells respond to injuries by phagocytosing cell debris in concert with peripheral macrophages (Jessen and Mirsky 2016). In addition, satellite glia in the developing peripheral ganglia phagocytose apoptotic neurons, likely via the engulfment receptors Jedi‐1 (PEAR1) and MEGF10 (Wu et al. 2009). In vitro, OECs have a higher capacity than Schwann cells for phagocytosis and produce lower amounts of pro‐inflammatory cytokines (Nazareth et al. 2020), but nothing is known with regard to the receptors involved.

The transcriptional profile of cultured OECs was found to be highly similar to Schwann cells (Franssen et al. 2008; Ulrich et al. 2014; Vincent et al. 2005), with gene expression differences found to be associated with tissue repair (Franssen et al. 2008). Single‐cell RNA‐sequencing of dissociated olfactory bulb (Tepe et al. 2018) and of cultured OECs from the olfactory bulb (Phelps et al. 2025) revealed five diverse OEC subtypes. OEC‐expressed genes show substantial overlap with those of Schwann cells, but also CNS glia such as microglia, astrocytes, and oligodendrocytes (Phelps et al. 2025). Despite similar characteristics of OECs and Schwann cells, the constant support of regeneration of the olfactory nerve is unique to OECs. Thus, OEC transplantation appears to be a promising treatment option for injuries of the nervous system (Murtaza et al. 2022).

OECs were shown to promote regeneration of the CNS in spinal cord injury models (Gómez et al. 2018; Khankan et al. 2016; Li et al. 1997; Richter et al. 2005). OECs secrete proteases to decrease the size of the glial scar (Pastrana et al. 2006; Simón et al. 2011), as well as neurotrophic factors and cytokines (Pastrana et al. 2007; Woodhall et al. 2001). While several studies showed promising results, outcomes are variable and the method needs improvement to enhance clinical success. Many transplantation studies were performed with OECs from the olfactory bulb. Olfactory bulb OECs express genes involved in the support of axon regeneration; olfactory mucosa OECs‐expressed genes were associated with the defense response, inflammation, and immunomodulation (Lan et al. 2022). Nevertheless, OECs from the lamina propria have exceptional translational potential due to the possibility of obtaining the cells from the olfactory mucosa of patients by biopsy (Holbrook et al. 2016), avoiding post‐transplant rejections. However, due to the limited number of OECs that can be harvested from human biopsies and the difficulty in ensuring a pure cell population of cultured OECs, the clinical outcome is not satisfactory. Therefore, a detailed understanding of OEC biology is essential to improve neural injury treatment.

We characterized here the activation of OECs upon neuronal injury. Comparison of steady state and injury response to other peripheral nervous system glia revealed marked similarities to satellite glial cells. We also show that OECs act in concert with perineural fibroblasts and characterize markers that can be used to distinguish between both cell types, which improves our understanding of OEC biology and helps to more precisely analyze cultured cells from the olfactory mucosa.

## 2 Methods

### 2.1 Animal Breeding and Treatment

Animal experiments were conducted in compliance with the EC directive 86/609/European Economic Community guidelines for animal experiments and authorized by the local government (Thüringer Landesamt für Lebensmittelsicherheit und Verbraucherschutz). The mice were maintained under a 12‐h light/dark cycle and had unrestricted access to food and water. C57BL6/6J wild‐type mice were initially acquired from Charles River Laboratories (Sulzfeld, GER). Tg(Mpz‐Cre)26Mes mice were procured from Charles River Laboratories (Sulzfeld, GER), where the Cre recombinase gene was controlled by a mouse Mpz (myelin protein zero) promoter (Feltri et al. 1999), with MPZ expressed in OECs and sustentacular cells. In regeneration experiments, 8 W (weeks) old mice were injected intraperitoneally with 50 mg/kg of methimazole (Sigma‐Aldrich, St. Louis, MO) and were euthanized with an overdose of isoflurane at 1, 3, 14, 42, or 92 days post‐injection (dpi).

### 2.2 Tissue Preparation and Immunofluorescence

The nasal tissues, ischiadic nerves, and dorsal root ganglia were fixed in 4% paraformaldehyde (PFA; Carl Roth, Karlsruhe, GER) at 4°C for 24 h. After cryopreservation in 30% sucrose and freezing in 2‐Methylbutane (Carl Roth, Karlsruhe, GER), tissues were embedded in tissue freezing medium (Leica Microsystems, Wetzlar, GER) on a specimen disk and sectioned coronally at a thickness of 18 μm. Antigen retrieval was performed in 0.1 mol/L citrate buffer (0.1 mol/L tri‐sodium‐citrate‐dehydrate, 0.5% Tween‐20, pH 6) at 97°C–99°C for 15 min. Slides were blocked in TBS Plus (2% Bovine Serum Albumin [BSA; Thermo Fisher Scientific Germany Ltd. & Co KG, Bonn, GER], 0.1% Triton in TBS), and incubated with primary antibodies and secondary antibodies (Table S1).

For quantification, three to seven animals from different litters kept in the same housing conditions were analyzed per group; sections were stained in parallel, and images taken for quantification were taken with the same microscope (TCS SPE system [Leica DM2500, Leica Microsystems, Wetzlar, GER] or Zeiss LSM900 equipped with Airy‐Scan technology). Measurements of areas and cell counts were performed on digital pictures using LAS X, ImageJ, or ZEN 3.0. Four regions of the septum were examined to determine cell counts. The background intensity in cells that do not express the protein of interest based on visual inspection was measured, and cells with intensity values at a minimum of five times the background value were counted as positive. Intensity measurements were conducted using ZEN 3.0 or LAS X; integrated density values were measured in regions of interest from six different areas per stained cryosection. Four to six sections were quantified and averaged. Data from each animal were grouped and used for statistical analysis; a minimum of three different mice were analyzed per condition. Images were further processed using LAS AF (Leica Microsystems), Image J, Photoshop CS6 (Adobe Systems, CA, USA), and ZEN 3.0 (blue edition) (Carl Zeiss Microscopy GmbH, Oberkochen, GER).

### 2.3 Alcian Blue Staining

Tissue sections were washed 3 times for 10 min in distilled water, incubated with Alcian blue (Sigma‐Aldrich, St. Louis, MO) for 2 min, and washed under running tap water for 5 min. Sections were rinsed with distilled water, dehydrated in ethanol, incubated in Xylene for clearing, and mounted in Entellan.

### 2.4 Transmission Electron Microscopy

OE from the septum was fixed with 4% PFA and 2.5% glutaraldehyde in cacodylic buffer (0.1 M sodium cacodylate, pH 7.2) for 2 h at RT and 4°C overnight. The tissue was stained with 1% osmium tetroxide for 1 h, dehydrated with increasing ethanol concentrations (30%, 50%, 70%, 80%, 90%, and 100%) accompanied by uranyl acetate staining at 50% ethanol, followed by infiltration of the tissue with a graded propylene oxide/epoxy resin (Araldite) series (2:1, 1:1, 1:2). The tissue was embedded in molds with epoxy resin and cured for 72 h at 60°C. The embedded tissue was trimmed, ultra‐thin (60 nm) sectioned using a Leica Ultracut S (Leica, Wetzlar, Germany), and stained with lead citrate. Finally, the specimens were studied in a transmission electron microscope (EM 900, Zeiss, Oberkochen, Germany) at 80 kV and a magnification of 20,000×. Imaging was done by exposing negatives.

### 2.5 RNA Sequencing and Transcriptomic Analysis

Three epithelia of WT mice were combined to form each sample. mRNA was isolated from control WT mice (8 W), at 3dpi and at 14dpi. RNA was isolated and transcriptional sequencing was performed on NovaSeq6000 systems by CeGaT GmbH (Tübingen, GER). RNA read counts obtained from high‐throughput RNA sequencing were analyzed for differential expression using the R Bioconductor software package DESeq2 (Love et al. 2014). The dataset was processed following the standard workflow provided and filtered based on an adjusted *p* value cutoff of 0.05. To determine histological localization and average expression under steady state conditions, differentially expressed genes were identified using a published RNA single‐cell sequencing dataset GSE169011 (Wang et al. 2022). Upregulated genes with a fold change (FC) greater than 1.3 underwent gene ontology (GO) term enrichment analysis using the ShinyGo (Ge et al. 2019). The results were visualized using the included dotplot function. Violin plots of genes expression were done with Loupe browser 7 (10X Genomics, Leiden, the Netherlands).

### 2.6 Quantitative Real‐Time PCR

Total RNA from 8 W old wild‐type mice (control, 3dpi, 14dpi, 42dpi, 92dpi) was extracted from olfactory mucosa samples using the Purelink RNA Mini Kit (Thermo Fisher Scientific Germany Ltd. & Co. KG, Bonn, GER); cDNA was generated using the High Capacity cDNA Kit (Thermo Fisher Scientific Germany Ltd. & Co. KG, Bonn, GER). Quantitative Real‐Time PCR (qPCR) was conducted on a Quant Studio 3 Real Time PCR Cycler (Thermo Fisher Scientific Germany Ltd. & Co. KG, Bonn, GER) using predesigned Quantitect primers (Qiagen N.V., Hilden, GER) for *Gapdh* (QT01658692), *Col11a1* (QT01055418), *Sparc* (QT00161721), *Bcan* (QT00105441), *Fasn* (QT00149240), *Fads1* (QT00114184), *Apoe* (QT01043889), *Acsbg1* (QT00108171), and *Hmgcs1* (QT00132461), along with Power Up SYBR Cyan Master Mix (Thermo Fisher Scientific Germany Ltd. & Co. KG, Bonn, GER). Three independent qPCR runs for each sample were performed. The qPCR conditions consisted of 2 min at 50.0°C, 2 min at 95.0°C, 44× 15 s at 95°C, followed by 1 min at 55°C, 15 s at 95°C, 1 min at 60°C, and 15 s at 95°C. Expression levels of mRNA were determined using the ΔΔCT method and displayed as 2^−ΔΔCT^ values (Schmittgen and Livak 2008).

### 2.7 Statistical Analysis

We conducted statistical analysis using Microsoft Excel and GraphPad Prism, representing the data as mean ± standard error of the mean (SEM). We assessed data for normal distribution and variance homogeneity. Statistical significance was established at *p < 0.05 and analyzed using either Student's *t*‐test.

## 3 Results

### 3.1 OECs Are in Close Contact With the Axons From Immature Neurons

**FIGURE 1:**
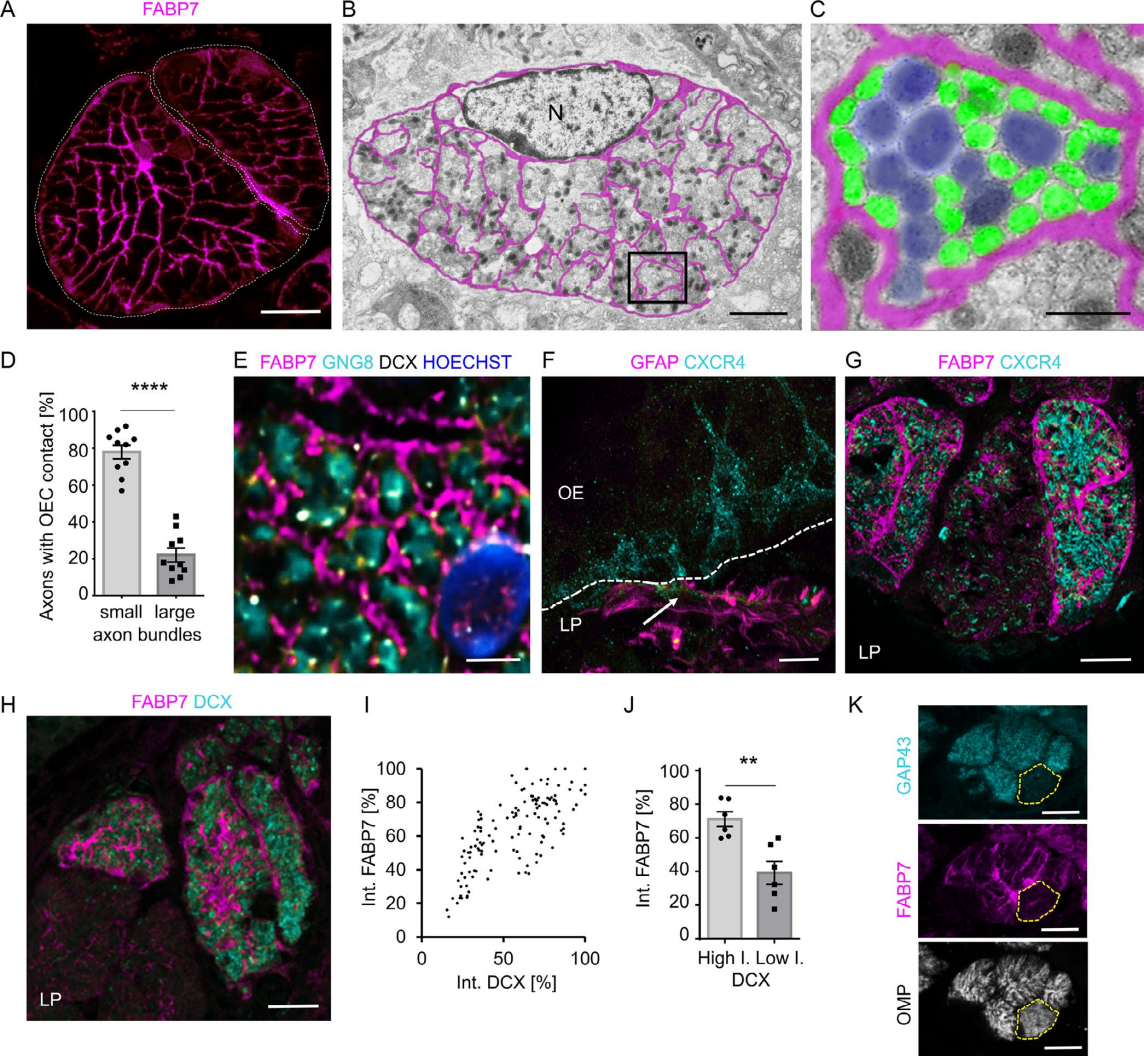
OECs are in close proximity to axon from immature neurons. (A) Immunofluorescence staining of FABP7 in WT mice, fine dotted lines represent the limitation of the axon bundles. (B) Electron microscopy of a small axon bundles with a single discernible OEC nucleus (N). The OEC extends membrane processed throughout the axon bundle (highlighted magenta for better visibility). (C) Higher magnification of the area indicated in B, OEC membrane extensions are labeled in magenta, large axons (> 200 nm) are labeled blue, small axons (< 200 nm) green. (D) Quantification of direct contacts between large and small axons with OECs (*n* = 10 axon bundles analyzed). (E) High power picture of axon markers GNG8 (cyan) and DCX (white) with the OEC marker FABP7 (magenta), nuclei are labeled with Hoechst (blue). (F) Labeling of OECs (GFAP, magenta) and nascent axons (CXCR4, cyan) showing close association (arrow). Dotted line represents the basal lamina. OE olfactory epithelium, lamina propria (LP). (G, H) Larger bundles surrounded by OECs (FABP7, magenta) and nascent axons (G, CXCR4, cyan) and axons from immature neurons (H, DCX, cyan). (I) Correlation of DCX and FABP7 intensities. (J) Significantly brighter FABP7 staining in bundles with axons from immature neurons (high DCX intensity) (*n* = 194 axon bundles). (K) Staining of the OEC marker FABP7 (magenta), immature neuron marker GAP43 (cyan) and mature neuron marker OMP (white), a segment containing only OMP‐positive axons (dotted line) is weakly labeled with FABP7. Student's *t*‐test, error bars represent SEM, **p < 0.01, ****p < 0.001. Scale bars (A) 20 μm, (B) 2 μm, (C) 0.5 μm, (E) 2 μm, (F–H, K) 10 μm.

OECs surround axon bundles passing through the nasal lamina propria into the CNS, acting as an important glial component of the olfactory nerve. In general, olfactory bulb OECs are more closely studied compared to mucosa OECs, although mucosa OECs are much easier accessible by biopsy and therefore have higher potential for patient use. We therefore aimed at analyzing mucosal OECs to understand their role in neuronal regeneration. OECs have thin laminar processes that wrap around the olfactory nerves and extend processes throughout the axon bundles. The network of OEC extensions can be observed in immunofluorescence staining of FABP7 (fatty acid binding protein 7) (Figures 1A and S1B) and by electron microscopy (Figure 1B). FABP7, also called BLBP (brain lipid binding protein), is expressed by radial glia cells and astrocytes of the CNS, but in the olfactory system serves as a specific marker of OECs (Kurtz et al. 1994). When analyzing the axons that are in direct contact with the OECs compared to axons located more inside the axon bundles, we observed differences in their diameter. Axons that are inside the bundle have a larger diameter (diameter 0.38–0.85 μm, colored blue in Figure 1C,D), while axons that are in direct contact with the OECs are smaller (diameter 0.14–0.3 μm, colored cyan in Figure 1C,D). When olfactory axons exit the outer nerve layer and lose contact with OECs, the axon diameter increases (Akins and Greer 2006; Au et al. 2002), indicating that axon diameter and OEC contact are correlated.

Staining for GNG8 (G protein gamma 8), a gene that is expressed already by cells in early stages of neurogenesis (immediate neuronal progenitor cells), was closely associated with FABP7 (Figure 1E). We also found a close association of GFAP (glial fibrillary acidic protein)‐positive OEC extensions with CXCR4 (C‐X‐C chemokine receptor type 4), another marker for immediate neuronal progenitor cells (Figure 1F). Moreover, OECs surrounding axon bundles with CXCR4‐positive axons showed overall intense labeling with FABP7 (Figure 1G). Axon bundles with high numbers of immature neurons, judged from expression levels of DCX (doublecortin), showed intense FABP7 labeling (Figure 1H). Quantification of FABP7 and DCX labeling in axon bundles revealed a positive correlation (Figure 1I, *r* = 0.72). Analysis of FABP7 intensities in bundles with high (> 50% of the intensity of the most intense labeled axon bundle) and low (< 50% of the intensity of the most intense labeled axon bundle) DCX staining intensity, respectively, also showed increased FABP7 staining in OECs surrounding axon bundles with DCX‐positive axons (Figure 1K). Bundles mostly containing axons labeled with OMP (olfactory marker protein), a marker for mature olfactory neurons, showed lesser FABP7 staining compared to bundles mostly composed of GAP43 (Growth‐associated protein 43)‐positive immature neurons (Figure 1L). Cell‐to‐cell variation in expression levels of *Fabp7* was also present in published single‐cell sequencing data (Figure S1C) (Wang et al. 2022).

Axons in bundles differ in their maturation state due to the ongoing turnover of the olfactory sensory neurons. FABP7 expression heterogeneity thus may be explained by variations in the signals that arrive from axons in different maturation stages (Fields and Stevens‐Graham 2002).

### 3.2 OECs Are Unique Glia Cells With Similarities to Satellite Glia Cells

**FIGURE 2:**
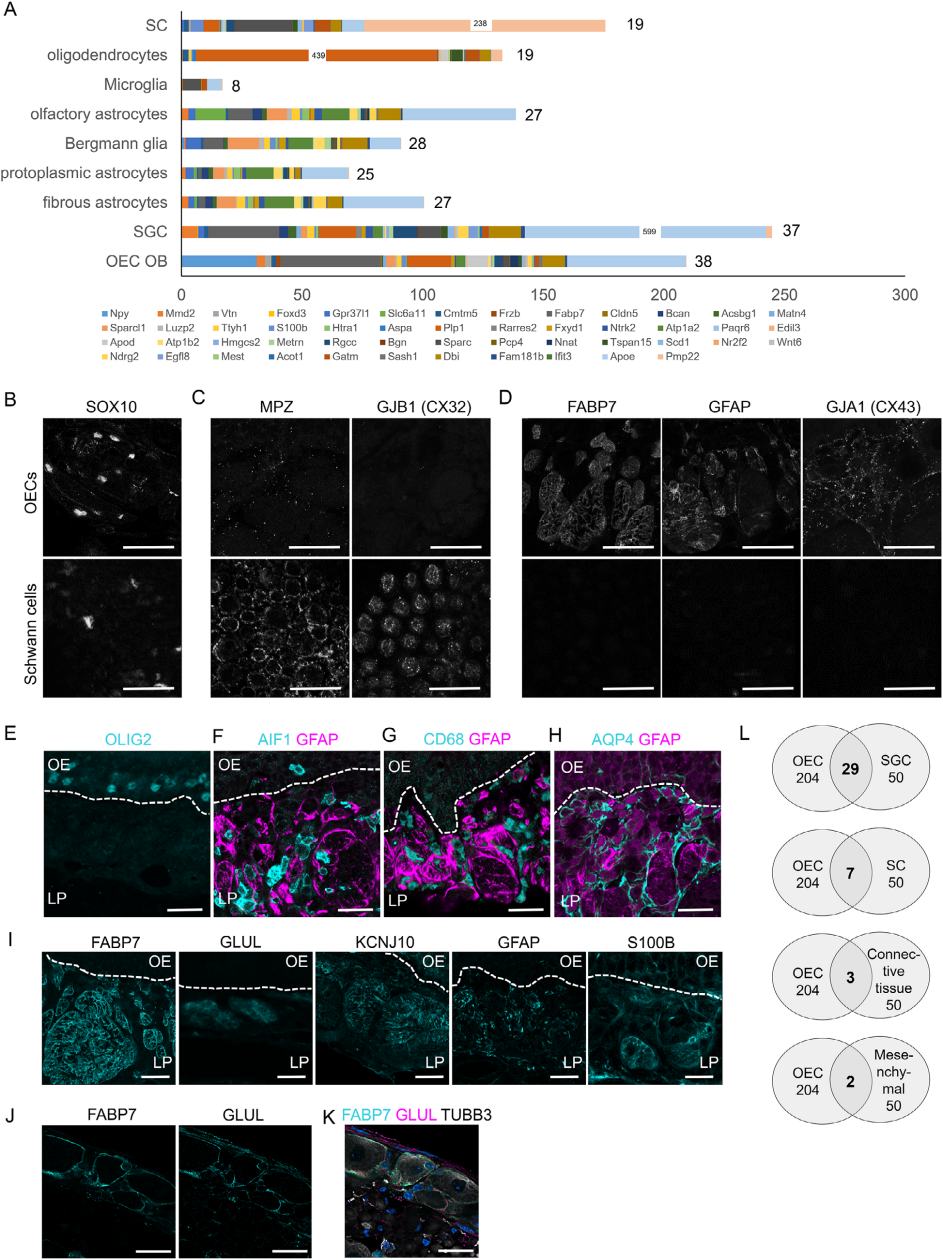
Olfactory ensheathing cells are different from other glia. (A) The 50 most specific OEC expressed genes were obtained from single cell transcriptomic data (Wang et al. 2022) and their expression was analyzed in glia cells from the peripheral and from the CNS (mousebrain.org). The bars represent the expression level in the respective cell type; Bars for three genes (*Apoe* in SGC, *Pmp22* in SC, *Plp1* in Oligodendrocytes) were shortened due to extremely high levels, true values are indicated as numbers in the respective bars. Total number of genes expressed in the respective cell type is depicted at the end of each bar. Immunofluorescence staining of (B) SOX10 in OECs of the lamina propria and in Schwann cells from the sciatic nerve. (C) Schwann cell markers MPZ and GJB1 in OECs and Schwann cells. (D) OEC markers FABP7, GFAP, and GJA1 in OECs and Schwann cells. (E) The oligodendrocyte marker OLIG2 is expressed by basal cells of the olfactory epithelium, but not in the lamina propria. Missing co‐localization of (F) GFAP (magenta) and AIF1 (cyan), (G) GFAP (magenta) and CD68 (cyan) in OECs of the LP. (H) AQP4 is expressed by Bowmann glands of the lamina propria, but did not co‐localize with GFAP (magenta). (I) Localization of satellite glia cell markers FABP7, GLUL, KCNJ10, GFAP, S100B in the lamina propria. (J) Localization of FABP7 and GLUL in satellite glia cells of the dorsal root ganglion. (K) Overlay of FABP7 (cyan) and GLUL (magenta) showing colocalization in satellite glia cells, neurons are labeled with the axonal marker TUBB3 (white), nuclei are labeled with Hoechst (blue). (L) The 50 most cell type specific genes were extracted from single cell transcriptome dataset obtained from the dorsal root ganglion (Avraham et al. 2021). Marker genes for satellite glia cells (SGC), Schwann cells (SC), connective tissue and mesenchymal cells were tested for expression in the OEC transcriptome, the overlap is depicted as Venn diagram. The overlap is strongest when comparing OECs to SGC. Dotted lines represent the basal lamina. OE olfactory epithelium, LP lamina propria. Scale bars 20 μm.

OECs are described to share similarities to other peripheral glia such as Schwann cells (Ulrich et al. 2014), but also express common genes with CNS astrocytic glia cells (Barber and Lindsay 1982). We therefore aimed at comparing gene expression between mucosal OECs and other glia cells. OEC expressed genes were extracted from a recent single cell sequencing study of the olfactory mucosa (Wang et al. 2022) and displayed as UMAP and violin plots (Figures S1 and S2). The 50 most OEC‐specific genes were compared to single cell sequencing data of other glia cells derived from the mouse brain atlas of cell types (mousebrain.org) (Zeisel et al. 2018). We compared mucosal OEC genes to OECs from the olfactory bulb, microglia, satellite glia cells, Schwann cells, different types of astrocytes (olfactory astrocytes, Bergmann glia, protoplasmic and fibrous astrocytes from the telencephalon) and mature oligodendrocytes (Figure 2A).

Mucosal OECs showed similarities to Schwann cells, but not more than to other glia cells with 19/50 shared genes and relatively low abundance of many genes in Schwann cells, except for highly expressed *Pmp22* (peripheral myelin protein 22, PMP22). PMP22 is not necessary for myelin formation in OECs, but may be involved in the regulation of paracellular permeability and cell proliferation (Roux et al. 2005). In addition, both OECs of the lamina propria and Schwann cells surrounding the sciatic nerve express SOX10 (SRY‐box transcription factor 10), a transcription factor involved in the expression of myelination genes (Figure 2A,B). GJB1 (Gap junction protein beta‐1, connexin 32) and MPZ (Myelin protein zero) are expressed by Schwann cells, but are barely detectable in OECs by immunofluorescence staining (Figure 2C). Nevertheless, low‐level expression of MPZ is present in OECs, as we found mRNA expression (Figure S1F) and labeling around axon bundles in Tg(*Mpz*‐Cre); R26^CAG‐LSL‐tdT^ mice (Figure S1P). On the other hand, clear OEC markers GFAP, FABP7, and GJA1 (Gap junction protein alpha‐1, Connexin 43) were not detected in Schwann cells by immunofluorescence staining (Figure 2D).

Although PLP1 (Proteolipid protein 1) and SOX10 are abundantly expressed by OECs and oligodendrocytes (Ye et al. 2023), marked differences were found in the comparison to oligodendrocytes (19/50 shared genes) and the oligodendrocyte marker OLIG2 (oligodendrocyte transcription factor 2) was not detectable in the lamina propria (Figures 2A,E and S2B). Minor similarities were found to microglia cells (8/50 shared genes, all at relatively low expression levels), and the microglia/macrophage markers AIF1 (allograft inflammatory factor 1, IBA1) and CD68 did not co‐localize to the OEC marker GFAP in the lamina propria (Figures 2F,G and S2B). OECs have higher similarities to astrocytes, that is, the abundant expression of *Apoe* (Apolipoprotein E) and *Gfap* (Figure 2A), but do not express the astrocytic marker protein AQP4 (aquaporin 4), which was found only in Bowmann glands of the lamina propria (Figure 2H).

Most parallels in gene expression were observed to OECs from the olfactory bulb (38/50 shared genes) and, unexpectedly, to satellite glia cells (37/50 shared genes) (Figure 2A). The OEC marker FABP7 has recently been described as a marker distinguishing satellite glia cells from Schwann cells in sensory ganglia (Avraham et al. 2021). We therefore analyzed other markers that distinguish satellite glia cells from Schwann cells and found that OECs indeed express all tested satellite glia cell‐specific markers (GLUL (Glutamate‐ammonia ligase), KCNJ10 (Potassium inwardly rectifying channel subfamily J member 10, Kir4.1), S100B (S100 calcium binding protein B)) (Figure 2I,K,L). When analyzing the 50 most abundant genes from satellite glia cells, Schwann cells, connective tissue, and mesenchymal cells of the dorsal root ganglion (https://mouse‐drg‐injury.cells.ucsc.edu/, (Avraham et al. 2021)), we found that the overlap between OEC and satellite glia cells is much stronger compared to Schwann cells (Figure 2M).

Taken together, OECs seem to be a unique type of glial cells, but showed the highest similarities to satellite glial cells when compared to other types of glia.

### 3.3 Olfactory Ensheathing Cells Respond to Methimazole‐Induced Injury

**FIGURE 3:**
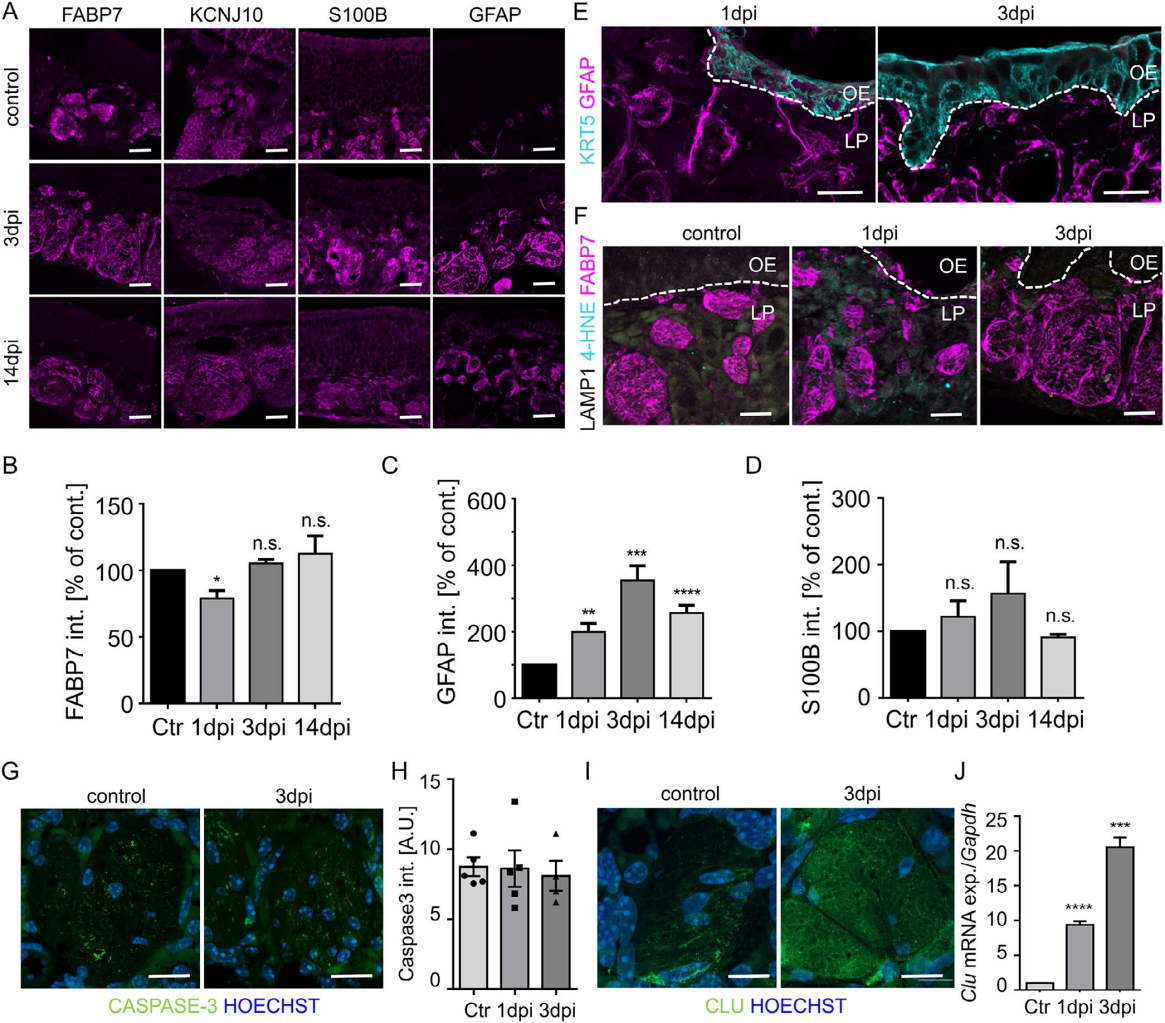
OECs respond to methimazole induced injury. (A) Representative immunofluorescence staining of OEC markers FABP7, KCNJ10, S100B and GFAP in OECs of control WT mice (8 W) and after injury (3 days post injection (dpi) and 14dpi). (B) Quantification of the FABP7 staining intensity (*n* = 3–6 mice). (C) Quantification of the intensity of GFAP (*n* = 3–7 mice). (D) Quantification of the intensity of S100B (*n* = 3–4 mice). (E) Immunofluorescence staining of the OEC marker GFAP (magenta) and the horizontal basal cell marker KRT5 (Keratin 5) (cyan), showing multiple sites of contact after injury. (F) Co‐staining of the OEC marker FABP7 (magenta), 4‐Hydroxynonenal (4‐HNE, cyan) as marker for oxidative stress and LAMP1 (white) as marker for lysosomal activity. (G) Localization of CASPASE‐3 (green) in axon bundles is not increased after injury, nuclei are stained with Hoechst (blue). (H) Quantification of CASPASE‐3 fluorescence intensities showing similar levels after injury (*n* = 3 animals per group). (I) Localization of Clusterin (CLU) (green) in axon bundles is increased shortly after injury (3dpi), nuclei are stained with Hoechst (blue). (J) Quantification of *Clu* mRNA transcripts by qPCR shows significant increase of *Clu* expression upon injury (*n* = 3 animals per group). Student's t‐test, error bars represent SEM, *p < 0.05, **p < 0.01, ***p < 0.001, ****p < 0.0001. Scale bars (A, E, F) 20 μm, (G), (I) 10 μm. Dotted line represents the basal lamina. OE olfactory epithelium, LP lamina propria.

Application of the thyroid drug methimazole destroys the olfactory epithelium due to the formation of toxic intermediates and is used to observe the processes associated with the formation of new neurons. Shortly after application of methimazole, the complete neuroepithelium detaches from the horizontal basal cells sitting on top of the basal lamina, possibly secondary to damage of Bowmann glands and sustentacular cells (Bergström et al. 2003). The axons of the sensory neurons remain in the lamina propria but are disconnected from their cell bodies. Three‐days post injury (dpi) horizontal basal cells proliferate to replenish the cells of the epithelium. In this early phase, we observed marked upregulation of GFAP in OECs of the lamina propria, whereas expression levels of OEC markers FABP7 and KCNJ10 did barely change (Figure 3A–C). S100B, a calcium‐binding protein with neurotrophic functions, was increased, but due to marked variability of the staining did not reach significance levels (Figure 3A,D). After 14 days, when many immature neurons are present and extend their axons towards the olfactory bulb, S100B expression already declined back to baseline (Figure 3A,D), while expression levels of GFAP stayed elevated (Figure 3A,C). OECs underwent marked morphological changes immediately after dissociation of the epithelium (1dpi, 3dpi); GFAP‐positive long processes extended to contact the proliferating horizontal basal cells (Figure 3E). Somewhat unexpectedly, disconnection of the axonal compartments from the neuronal cell bodies did not cause major oxidative stress in the lamina propria, as detected by (absent) 4‐Hydroxynonenal staining (Figure 3F, 4‐HNE, cyan). Although OECs have been described to phagocytose axonal debris (Nazareth et al. 2015), we detected upregulation of the lysosomal marker LAMP1 (lysosomal associated membrane protein 1) in horizontal basal cells and few dispersed cells in the lamina propria but not in FABP7‐positive OECs (Figure 3F, white). Moreover, labeling of the lamina propria with the apoptosis marker CASPASE‐3 was indistinguishable between control conditions and after injury (Figure 3G,H). Consistent with absence of detectable cell stress, we observed upregulation of Clusterin (CLU) (Figure 3I,K), a secretory protein that protects astrocytes from oxidative stress (Sultana et al. 2025). Clusterin was localized inside of the axon bundles of the lamina propria, in agreement with a function of amelioration of cell stress. Although Clusterin is not exclusively expressed by OECs (Figure S2A), other potentially secreting cell types are either not present after degradation of the epithelium (sustentacular cells, neurons) or do not secrete (Bowmann glands).

### 3.4 Olfactory Ensheathing Cells Do Not Proliferate After Methimazole Induced Injury

Not many studies so far have investigated if OECs proliferate after injury. Unilateral bulbectomy led to a transient, small increase in EdU‐positive OECs in the olfactory mucosa of newborn mice (P4.5), when OECs migrate from the peripheral olfactory nerve to populate the empty cavity (Chehrehasa et al. 2012). On the other hand, the loss of olfactory neurons caused by intracranial sectioning of the olfactory nerves did not result in the division of OECs (Li et al. 2005). In addition, ZnSO4 inhalation induced degeneration of the olfactory epithelium and a marked inflammatory response in both the epithelium and the bulb, but did not result in OEC proliferation (Williams et al. 2004). Moreover, axon packing and OEC cell density in the olfactory nerve bundles in the lamina propria remained almost constant from 10 days through 16 months of age, in contrast to reported data on other glial cell types in the peripheral and CNSs (Watanabe et al. 2006).

**FIGURE 4:**
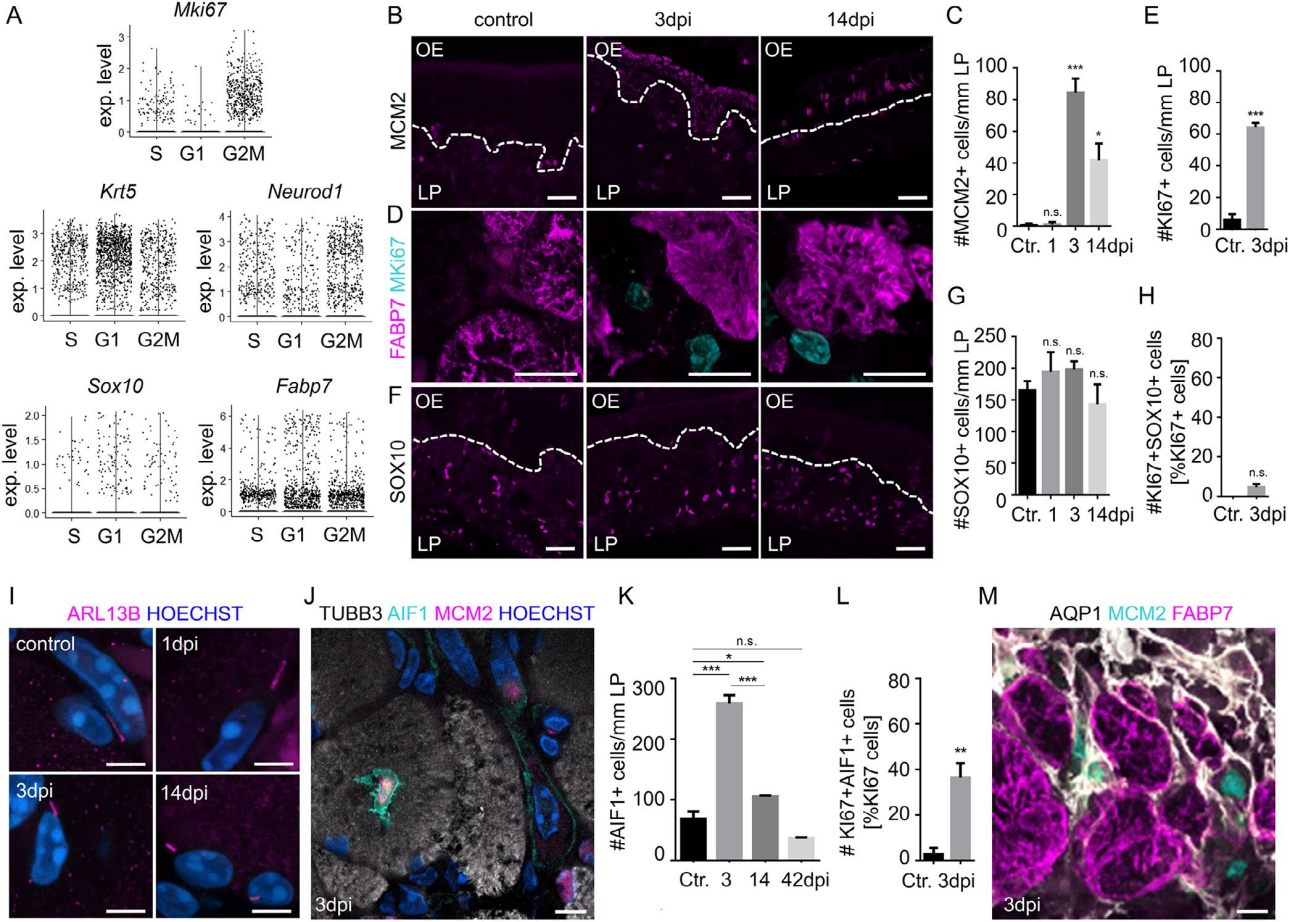
OECs do not proliferation upon injury. (A) Cell cycle scores were assigned to cells from published single cell RNA sequencing data (Wang et al. 2022) based on expression of G2/M and S phase markers using R package for cell cycle classification. Cell cycle scoring for Mki67 expressing cells showed G2M enrichment (positive control). Analysis of quiescent Krt5‐positive horizontal basal cells showed G1 enrichment, analysis of Neurod1 expressed by proliferating neuronal progenitors showed G2M enrichment. OEC, identified by Sox10 and Fabp7 expression, did not show enrichment in G2M phase, showing that the cells do not proliferate under steady state conditions. (B) Staining of the proliferation marker MCM2 in the lamina propria. (C) Quantification of the number of MCM2‐positive nuclei in the lamina propria (*n* = 3 mice). (D) Absence of co‐localization between OEC marker FABP7 (magenta) and MKI67 (cyan) in the lamina propria. (E) Quantification of the number of MKI67‐positive nuclei in the lamina propria (*n* = 3 mice). (F) Staining of OEC marker SOX10 in the lamina propria. (G) Quantification of the number of SOX10‐positive nuclei in the lamina propria showing no significant difference between control and post‐injury conditions (*n* = 3 mice). (H) Quantification of the number of SOX10‐positive nuclei among all MKI67‐positive cells in the lamina propria showing that only very few OECs proliferate, differences are not significant (*n* = 3 mice). (I) Presence of cilia marked by ARL13 (magenta) at nuclei (Hoechst, blue) of OECs in control mice and during regeneration. (J) Co‐localization of macrophages (AIF1, cyan) with the proliferation marker MCM2 (magenta) inside of an axon bundle (labeled with TUBB3, white), nuclei are labeled with HOECHST (blue). (K) Quantification of the number of AIF1‐positive cells in the lamina propria (*n* = 3 mice). (L) Quantification of the number of AIF1‐positive nuclei among all MKI67‐positive cells in the lamina propria, showing that macrophages/monocytes proliferate (*n* = 3 mice). (M) Co‐localization of MCM2 (cyan), FABP7 (magenta) and the fibroblast marker AQP1 (white) showing that some fibroblasts proliferate. Dotted lines represent the basal lamina. Student's t‐test, error bars represent SEM, *p < 0.05, **p < 0.01, ***p < 0.001, Scale bars (B, F), 20 μm, (D, J, M) 10 μm, (I) 5 μm. Dotted line represents the basal lamina. OE olfactory epithelium, LP lamina propria.

Due to conflicting results reported previously, we analyzed the cell cycle state of OECs (Wang et al. 2022) using R software. We tested *Mki67* (Marker of Proliferation Ki‐67)‐positive cells and detected enrichment in the G2M phase of the cell cycle, as expected (Figure 4A). We found that *Sox10* or *Fabp7* positive OECs were not enriched in G2M phase, similar to *Krt5* expressing quiescent horizontal basal cells, and unlike proliferating *Neurod1*‐positive immediate neuronal progenitor cells, indicating that OECs do not proliferate at steady state (Figure 4A). Increased expression of the cell cycle marker MCM2 (minichromosome maintenance 2) (Figure 4B,C) and MKI67 (Figure 4D,E) revealed increased numbers of proliferating cells in the lamina propria after methamizole‐induced injury, but the number of SOX10‐positive OECs did not change (Figure 4F,G). MKI67, a protein that is associated with cellular proliferation, mostly stained nuclei outside of FABP7‐positive axon bundles (Figure 4D) and SOX10‐positive nuclei were only very rarely stained by MKI67 (Figure 4H). Moreover, OECs of the axon bundles maintained their cilia during regeneration, showing that the cells do not undergo the cell cycle (Figure 4I). Inside the axon bundles, the cell cycle marker MCM2 was mainly expressed by AIF1‐positive macrophages and/or monocytes (Figure 4K), and the number of macrophages in the lamina propria increased after injury (Figure 4L). In addition, the percentage of proliferating AIF1‐positive cells increased after injury (Figure 4M). Some of the proliferating cells in the vicinity, but not inside the axon bundles, express markers for fibroblasts (AQP1, aquaporin 1) (Figure 4N).

As a result, OECs appear not to undergo cell division following injury, which is another similarity to satellite glia cells and a difference from Schwann cells. In injury models of the dorsal root ganglion, cell proliferation occurred in macrophages, but not in satellite glia cells (Avraham et al. 2021; Jager et al. 2020), whereas injury‐induced regeneration induces Schwann cell dedifferentiation and proliferation (Chen et al. 2007).

### 3.5 Perineural Fibroblasts in the Lamina Propria Respond to Injury

Fibroblasts define the architecture of tissues, expand the function and positioning of other cell types, and have key roles in many diseases. Perineural fibroblasts of the lamina propria express NGFR (nerve growth factor receptor, also called p75NTR/p75 neurotrophin receptor) and enwrap olfactory axon fascicles consisting of axons and OECs (Khan et al. 2022). In addition, lamina propria fibroblasts were shown to express ICAM1 (Intercellular adhesion molecule 1) and might function as mesenchymal stem cells (Delorme et al. 2010; Tomé et al. 2009).

**FIGURE 5:**
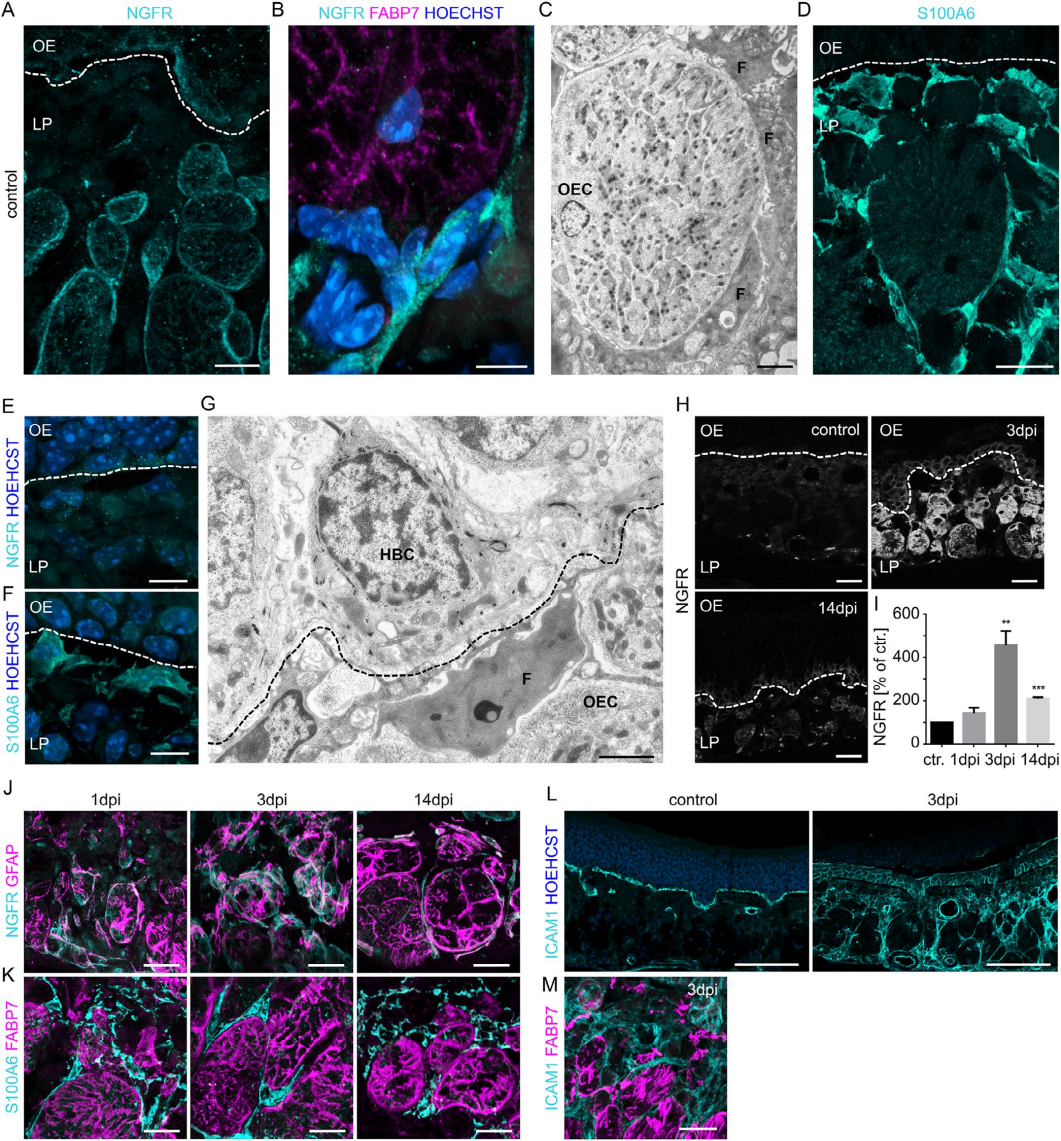
Different types of fibroblast in the lamina propria. (A) Staining of NGFR (cyan) in the lamina propria of control WT mice. (B) NGFR (cyan) and FABP7 (magenta) in the lamina propria did not co‐localize, showing that OECs do not express NGFR under control conditions. (C) Transmission electron microscopy picture showing fibroblasts (F) with electron dense cytoplasm surrounding axon bundles with OECs (electron‐lucent cytoplasm, OEC). (D) S100A6 (cyan) labels elongated perineural fibroblasts surrounding an axon bundle and fibroblasts in the vicinity of the basal lamina. (E) Fibroblasts at the basal lamina do not express NGFR, but (F) do express S100A6. (G) Transmission electron microscopy picture showing fibroblasts (F) with electron dense cytoplasm at the basal lamina (dotted line); the cells have many sites of contact with horizontal basal cells (HBC). (H) Representative immunofluorescence staining of NGFR in the lamina propria of control WT mice, 3 dpi and 14dpi. (I) Quantification of staining intensity showing an increase after injury (*n*=3‐4 mice, Student's t‐test, error bars represent SEM, **p < 0.01, ***p < 0.001). (J) Representative immunofluorescence staining showing co‐localization between OEC marker GFAP (magenta) and NGFR (cyan) during regeneration. (K) Fibroblasts and OEC during regeneration, S100A6 and FABP7 never co‐localized. (L) Immunofluorescence staining showing expression of ICAM1 in horizontal basal cells (control) and up‐regulation in fibroblasts during regeneration (3dpi), nuclei are stained with Hoechst (blue). (M) ICAM1 did not co‐localize with FABP7 (magenta), showing expression in perineural fibroblasts. Dotted lines represent the basal lamina. OE olfactory epithelium, LP lamina propria. Scale bars (A), (D), (J), (K), (N) 10 µm, (B), (E) 5 µm, (C), (G) 2 µm, (H) 20 µm, (L) 100 µm.

We tested fibroblast marker localization in the lamina propria and found NGFR staining mainly in cells surrounding axon bundles of the basal lamina propria (Figure 5A). In steady state conditions, NGFR was exclusively expressed by fibroblasts surrounding the axon bundle; the staining did not overlap with the OEC marker FABP7 (Figure 5B). This finding is highly relevant since antibodies that recognize NGFR are commonly used to isolate OECs from olfactory epithelial biopsies for cell transplantation. Electron microscopy showed a close association of fibroblasts with extensive filopodia‐like membrane protrusions, recognized by the dark cytoplasm, that surround an axon bundle composed of axons and OECs with pale cytoplasm (Figure 5C). NGFR‐positive fibroblasts were only present around the large axon bundles of the lamina propria, but not close to the basal lamina. We therefore tested S100A6 as an alternative fibroblast marker, since it was found to be expressed by olfactory bulb fibroblasts (Phelps et al. 2025). S100A6 was expressed by fibroblasts surrounding axon bundles, but also in cells close to the basal lamina, where NGFR staining was very weak or absent (Figure 5D–F). Electron microscopy analysis also showed the presence of electron‐dense fibroblasts close to the basal lamina (Figure 5G). Our findings of different fibroblast types in the lamina propria, associated with OECs and horizontal basal cells, respectively, are in accordance with recent single‐cell sequencing data showing that fibroblasts build a heterogeneous population of cells with distinct molecular signatures in many tissues (Buechler et al. 2021).

The close association of fibroblasts and OECs indicates a role of both cell types in the maintenance of the olfactory mucosa. Indeed, fibroblasts in the lamina propria responded to injury of the olfactory epithelium with massive upregulation of NGFR, peaking shortly after injury (Figure 5H,I). In the injury response, NGFR was not only detected in cells surrounding axon bundles, but it was also transiently found inside the axon bundles and in activated horizontal basal cells of the olfactory epithelium (Figure 5H), indicating that NGFR is part of a common injury response. We confirmed expression in OECs after injury by co‐localization of NGFR with GFAP (Figures 5K and S3A) and with FABP7 (Figure S3B). OECs and fibroblasts retain their steady‐state characteristics, as S100A6 and FABP7 never co‐localized (Figures 5L and S3C). Moreover, S100A6 was not expressed by cells inside the axon bundles, showing that fibroblasts do not enter the axon bundles (Figure 5L). As a result, both fibroblasts and OECs upregulate NGFR in response to olfactory epithelial injury; however, NGFR expression in steady‐state OECs was beyond the detection limit for immunofluorescence staining.

Upon injury, we observed morphological changes of fibroblasts surrounding the axon bundles, indicating activation of the cells (Figure 5K,L). Besides NGFR, fibroblasts upregulated the expression of ICAM1 upon injury (Figure 5M). In the non‐injured epithelium, ICAM1 is expressed predominantly in horizontal basal cells and blood vessels (Figures 5M and S3D). During regeneration, ICAM1 expression was markedly upregulated in the lamina propria (Figure 5M). Absence of co‐localization with GFAP proved expression in fibroblasts (Figures 5N and S3E,D). ICAM1 is markedly upregulated during differentiation of colonic fibroblasts to inflammatory cells and fibroblast–monocyte interactions (Matellan et al. 2024). Therefore, fibroblasts acquire a proinflammatory phenotype in injury‐induced regeneration that may help to recruit macrophages.

### 3.6 Fibroblasts, but Not OECs Express Proteins for Water Transport

Aquaporins are a family of water‐channel proteins that facilitate the transport of water and other small molecules across cell membranes. In the brain, several aquaporins have been identified, but AQP4 is the most abundant and plays a crucial role in facilitating water flow in response to potassium elevation in the extracellular space, thereby maintaining a stable ionic environment for neuronal signaling (Rash 2010). AQP4 is predominantly expressed in the foot processes of astrocytes associated with blood vessels, influencing the development and resolution of edema following traumatic brain injury.

**FIGURE 6:**
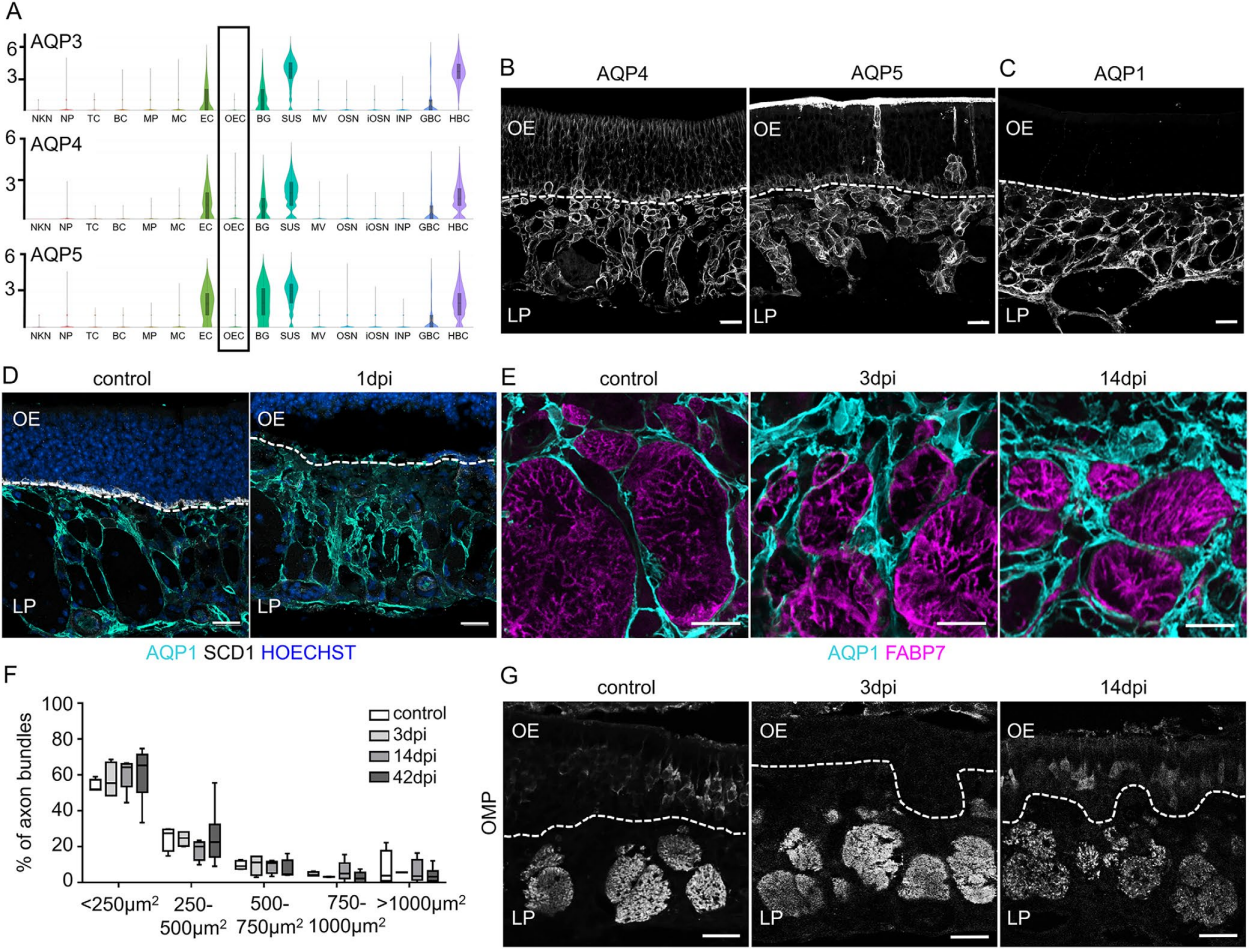
Aquaporin localization in the lamina propria. (A) Violin plots showing the expression of Aquaporin genes *Aqp3, Aqp4 and Aqp5* in different cell types of the olfactory epithelium, derived from published data (Wang et al. 2022). OECs do not express Aquaporin genes. (B) Representative immunofluorescence staining of AQP4 and AQP5 in Bowmann glands, sustentacular cells and horizontal basal cells. (C) Immunofluorescence staining of AQP1 in fibroblasts. (D) Comparison of staining of AQP1 (cyan) in control conditions and 1 day after injury (1dpi), horizontal basal cells are stained with Syndecan (SCD1, white), nuclei are stained with Hoechst (blue). (E) AQP1 (cyan) and FABP7 (magenta) did not co‐localize. (F) Quantification of axon bundle size in control mice, 3dpi, 14dpi, 42dpi, showing that the size did not change (axon bundles are grouped into bins, *n* = 3–6 mice, Student's *t*‐test, error bars represent SEM, all differences are not significant). (G) Example picture of axon bundles stained with OMP (control, 3dpi, 14dpi) showing similar sizes of axon bundles. Dotted lines represent the basal lamina. OE olfactory epithelium, LP lamina propria. Scale bars (B–D, G) 20 μm, (E) 10 μm.

Analysis of aquaporin expression in a single cell transcriptome of the olfactory epithelium (Wang et al. 2022) revealed very little expression of Aquaporin mRNA in OECs (Figure 6A, full set of *Aqp* genes expression in Figure S4A). *Aqp3*, *Aqp4*, and *Aqp5* are abundantly expressed in Bowmann glands, sustentacular cells, and horizontal basal cells; *Aqp11* is expressed only by Bowmann glands and sustentacular cells. As expected from transcriptome analysis, AQP4 and AQP5 were localized to Bowmann glands, horizontal basal cells, and sustentacular cells (Figure 6B). Similar to astrocytes, satellite glia cells of the dorsal root ganglion also expressed AQP4 (Figure S4B). We found Bowmann glands collapse after degeneration of the olfactory epithelium (Figure S4C), and the staining of AQP4 and AQP5 at 3dpi showed aggregates in the lamina propria, close to the basal lamina (Figure S4D,E). AQP1 is a constitutively open, bidirectional water channel expressed in fibroblasts (Gao et al. 2013). AQP1‐positive OECs have been described (Shields et al. 2010), but *Aqp1* mRNA is not expressed in OECs according to the transcriptomic analysis (Figure S4A). Instead, AQP1 was found in FABP7‐negative perineural fibroblasts surrounding the axon bundles (Figure 6C). In response to injury, we observed upregulation of AQP1 expression and a marked rearrangement of fibroblast morphology, especially in the initial injury response. In control conditions, fibroblasts were very thin, elongated cells that extend along the axon bundles; during regeneration, fibroblasts appeared swollen, possibly caused by increased water transport due to upregulation of AQP1 (Figure 6D). AQP1 expression stayed restricted to fibroblasts and was not expressed by OECs, independent of the condition (Figure 6E). In contrast, AQP1 was expressed in fibroblasts surrounding satellite glia cells in the dorsal root ganglion, but also in some FABP7‐positive satellite glia cells (Figure S4F). OECs did not obviously change their morphology upon injury (Figure 6D, see also Figure 4). Moreover, the size of the axon bundles in the lamina propria was the same in all stages of regeneration (Figure 6F,G), indicating that OECs maintain stable channels for axon outgrowth.

### 3.7 OECs Express Claudin 5, the Tight Junction Protein of the Blood Brain Barrier

**FIGURE 7:**
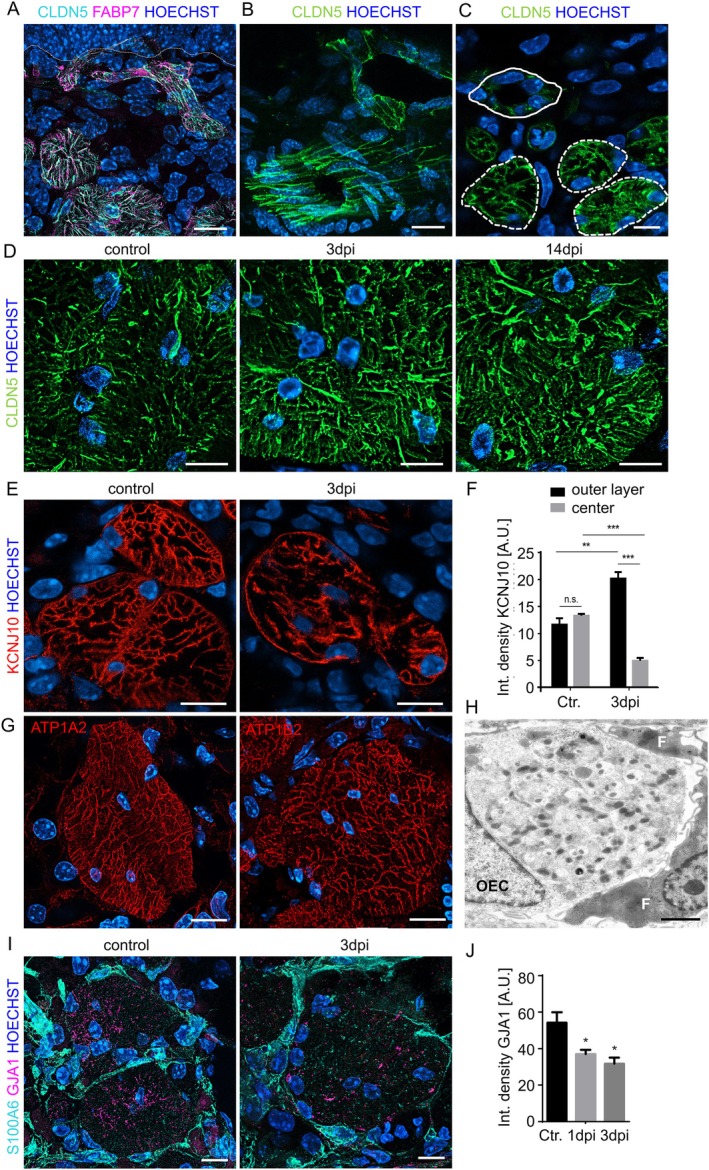
Cell junction proteins and electrolyte transporters in OECs. (A) Staining of CLDN5 (cyan) in OECs (FABP7‐positive, magenta) ensheathing axons passing the basal lamina (dotted line), nuclei are stained with Hoechst (blue). (B) OECs at the cribiforme plate form channel like structures, which are CLDN5‐positive (green). (C) CLDN5 labeling of small capillaries (solid circle) is weaker compared to OECs in axon bundles (dotted circles). (D) OECs in large axon bundles expressed CLDN5 (green), the staining was increased after injury. (E) Staining of KCNJ10 (red) in axon bundles showed localization on membranes extending through the axon bundle, but also on the OEC membrane lining the outer border. (F) Quantification of KCNJ10 staining on membrane compartments inside of the bundle compared to the outer membrane, showing that KCNJ10 shifted to the outer membrane at 3dpi (*n* = 3 mice). (G) Staining of ATP1A2 (left) and ATP1B2 (right) in OECs shows localization on membranes inside of the axon bundle, but not on the outer limiting membrane. (H) Transmission electron microscopy showing an axon bundle with an OEC and fibroblasts (F) with darker cytoplasm and membrane protrusions; OECs and fibroblast are in close vicinity, but separated by intercellular space containing collagen fibrils. (I) Staining of GJA1 (connexin 43, magenta) in axon bundles. GJA1 was localized inside of the axon bundles, but not detected between fibroblasts (cyan, S100A6) and OECs. Gap junctions were markedly reduced during after injury. (J) Quantification of GJA1 inside of axon bundles (*n* = 3 mice). Student's t‐test, error bars represent SEM, *p < 0.05, **p < 0.01, ***p < 0.001. Scale bars 10 μm, (H) 2 μm.

Having shown the marked stability of the OECs in the axon bundles, we became interested in genes that are special for OECs compared to other glial cells. Comparing OEC‐expressed genes (Wang et al. 2022) to other glial cells (derived from the mouse brain atlas of cell types (mousebrain.org) (Zeisel et al. 2018)) or from sequencing of the dorsal root ganglion (Avraham et al. 2021) revealed three genes unique to mucosal OECs: *Vtn* (vitronectin), *Cldn5* (Claudin 5), and *Frzb* (Frizzled related protein). FRZB, a Wnt‐binding protein and a competitor for the cell‐surface G‐protein receptor Frizzled, is required for olfactory axon targeting (Rich et al. 2018). The extracellular matrix glycoprotein VTN and the tight‐junction protein Claudin 5 are known to be exclusively expressed by vascular endothelial cells and pericytes and are required for the integrity of the blood–brain barrier. OECs in the outer nerve layer of the olfactory bulb contribute functionally to the blood–brain barrier (Beiersdorfer et al. 2020). No glial cell type, except OECs of the olfactory bulb, does express *Vtn* or *Cldn5* mRNAs (www.mousebrain.org). We therefore analyzed CLDN5 expression in OECs and glial cells of the dorsal root ganglion and found co‐localization with FABP7 in OECs (Figures 7A and S5A), but not in satellite glial cells or Schwann cells (Figure S5B,C). CLDN5 was not only expressed by OECs of large axon bundles, but also by OECs close to the basal lamina, building channel‐like structures that project from the olfactory epithelium into the deeper lamina propria (Figures 7A and S5A). Moreover, CLDN5 labeled OECs that envelop axon bundles transversing through the cribriform plate towards the olfactory bulb (Figure 7B). CLDN5 was also expressed by endothelial cells of small capillaries in the olfactory mucosa, but the expression level was higher in OECs (Figure 7C). Occludin, another common tight junction marker, was not detected in OECs (Figure S5D). The apical layer of activated horizontal basal cells, involved in shielding the regenerating epithelium, temporarily expressed CLDN5 after injury (Figure S5E). During injury‐induced regeneration, CLDN5 expression in OECs was stronger compared to control conditions (Figures 7D and S5F). OECs displayed marked CLDN5 expression, which indicates that they are important for shielding the axons that project directly into the CNS, representing a potential pathogen entry point.

If OECs shield the axons, the cells would have to maintain ionic homeostasis inside of the axon bundles. Inwardly rectifying potassium channels (Kir) are responsible for a high potassium permeability in satellite glia cells, relevant for maintenance of the resting membrane potential and indirectly for the neuronal resting potential (Olsen and Sontheimer 2008). After peripheral nerve injury, reduced expression of KCNJ10 (Kir4.1) and lower K^+^ buffering capacity leads to neuropathic pain (Vit et al. 2008), and K^+^ clearance via KCNJ10 in oligodendrocytes is critical for sustained axonal function and integrity (Schirmer et al. 2018). KCNJ10 was localized on OEC membranes extending through the axon bundles, but also in the OEC membrane lining the outer border of the bundle (Figure 7E). Upon injury, KCNJ10 shifted to the outside lining relative to the internal membrane compartments, indicating that KCNJ10 could be involved in extruding potassium from the axon bundles; overall expression levels of KCNJ10 did not decline (Figure 7E,F). Furthermore, OECs specifically express the β2 isoform of the Na^+^K^+^ ATPase (ATP1B2, AMOG), which is also promoting neuron–glia interactions and neurite outgrowth (Antonicek et al. 1987; Müller‐Husmann et al. 1993), in addition to the more ubiquitously expressed alpha 2 isoform ATP1A2 (Figure 7G). ATPase was co‐localized at internal membranes with FABP7 (Figure S5G). The OEC membranes lining the outer border of the axon bundle were devoid of ATPase (Figure 7G), suggesting they could be involved in taking up K^+^ released by neurons. Expression of ATP1B2 did not change markedly during injury‐induced regeneration. While OECs shield the axon bundle, the cells are connected via gap junctions (Piantanida et al. 2019). Direct contacts between OECs and fibroblasts were not observed by electron microscopy (Figure 7H). After injury, we found decreased levels of connexin 43 (GJA1) in OECs (Figure 7I,K). Blocking gap junctions has been shown to produce OEC hyperpolarization, possibly due to a shunting effect on KCNJ10 channels and a bigger impact of the KCNJ10 conductance on the resting potential (Rela et al. 2015). Gap junction degradation could therefore improve K^+^ homeostasis in OECs. Gap junctions were concentrated in the central part of the axon bundle, not at the interface between OECs and fibroblasts (Figures 7I and S5H), suggesting that gap junction communication occurs only among OEC.

### 3.8 OECs Upregulate Lipid Metabolism Genes in Response to Methimazole‐Induced Nerve Injury

**FIGURE 8:**
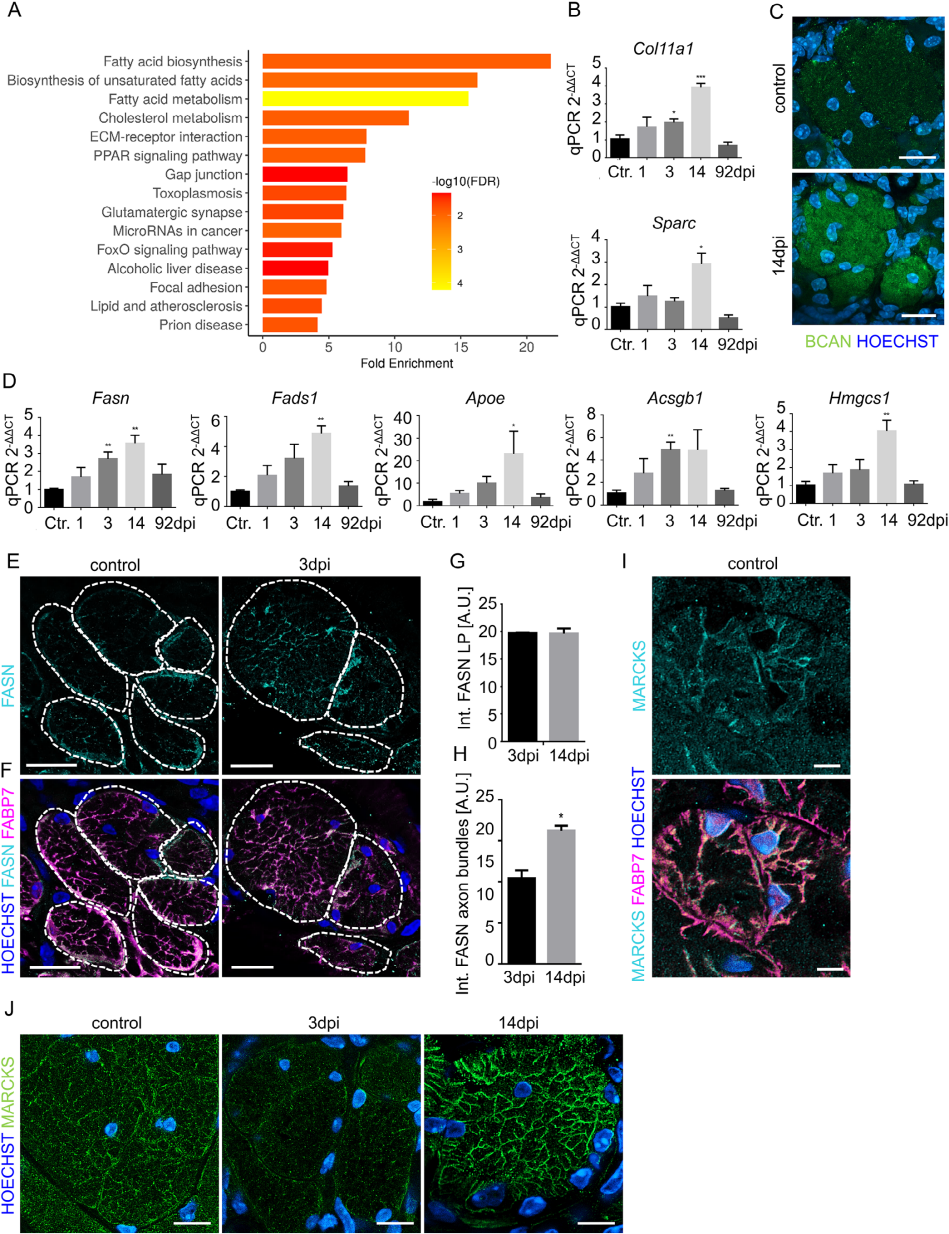
Upregulation of lipid metabolism genes in OECs during regeneration. (A) The transcriptome of the olfactory epithelium during methimazole‐induced injury was sequenced and subsequently mapped to the different cell types using a published single‐cell RNA sequencing dataset from murine olfactory mucosa (Wang et al. 2022) to identify genes specifically expressed by OECs. GO term enrichment for biological processes was performed for the OEC genes that were regulated during injury‐induced regeneration (14dpi), significance as determined by adjusted *p* values in the GO analysis is indicated by color. (B) Quantitative PCR for the matrix components *Col11a1* and *Sparc* from olfactory mucosa mRNA showing differences in post‐injury conditions (*n* = 3 animals per group). (C) Staining of Brevican (BCAN) at control, three and 14dpi showing increased intensities in axon bundles. Nuclei are stained with Hoechst (blue). (D) Quantitative PCR for lipid metabolism genes regulated in the transcriptome. Olfactory mucosa mRNA expression is analyzed relative to *Gapdh* mRNA in control and post‐injury conditions (*n* = 3 animals per group). (E, F) Co‐localization of FASN (cyan) and FABP7 (magenta) showing that both proteins redistribute to internal membranes of the axon bundle (encircled) during the axon extension phase of regeneration. (G) Quantification of the total staining intensity of FASN in the lamina propria, no difference was observed between injury conditions (*n* = 3 animals per group). (H) Quantification of FASN inside of the axon bundles revealed significant increase at 14 dpi. (*n* = 3 animals per group). (I) Redistribution of MARCKS to internal membranes of the axon bundle during the axon extension phase of regeneration (14dpi). (J) Staining of MARCKS (cyan) showing complete overlap with FABP7 (magenta) in the axon bundles. Nuclei are stained with Hoechst (blue). Student's t‐test, error bars represent SEM, *p < 0.05, **p < 0.01, ***p < 0.01. Scale bars (C), (I) 10 μm, (E, F) 20 μm, (J) 5 μm.

To define the transcriptional response of OECs to nerve injury, we analyzed the transcriptome of the olfactory epithelium under control and methimazole‐induced injury conditions using bulk RNA sequencing. We then specifically analyzed regulated genes in the OEC cluster by mapping the regulated genes to the different cell types in the olfactory epithelium based on published single cell data (Wang et al. 2022). Analysis for enriched biological pathways using KEGG analysis (Kyoto Encyclopedia of Genes and Genomes) revealed enrichment in lipid metabolic pathways, including fatty acid biosynthesis, as well as extracellular matrix and gap junctions (Figures 8A and S6A). The expression of selected regulated genes was confirmed in steady state OECs (Figures S2 and S6B).

Upregulation of the extracellular matrix components *Col11a1* (Collagen 11a1), *Sparc* (Secreted protein acidic and rich in cysteine), and *Bcan* (Brevican) was confirmed by quantitative PCR (Figures 8B and S6C). *Sparc* is able to promote axon sprouting and is involved in regulating plasticity and repair in the CNS. OECs are intensely immunopositive for SPARC during embryogenesis, but down‐regulate SPARC postnatally. Upregulation during injury (Figure 8B) therefore supports a role in helping neurite regrowth activity. BCAN was found distributed throughout the axon bundles, showing that OECs, similar to astrocytes (Hamel et al. 2005), secrete the protein to support incoming axons (Figure 8C). In addition, we observed a temporal degradation of the basal lamina during the injury response, but very rapid rebuilding already after 3 days (Figure S6D), showing marked rearrangement of the extracellular matrix during the injury response.

Quantitative PCR then confirmed increased expression of lipid metabolism genes *Fasn* (Fatty acid synthase 1), *Fads1* (Fatty acid desaturase 1), *Apoe* (Apolipoprotein E), *Acsbg1* (Acyl‐CoA Synthetase Bubblegum Family Member 1), and *Hmgcs1* (Hydroxymethylglutaryl‐Coenzym A‐synthase) during regeneration, with the highest expression observed during the phase of massive axonogenesis (14dpi) and a decline to baseline levels after completion of neurogenesis (Figure 8D). FASN, APOE, LRP1 (LDL receptor‐related protein 1), FABP7, and MARCKS were detected in axon bundles (Figure S6E). While FASN and APOE were mainly localized inside the axon bundle, LRP1 was also localized in structures surrounding the axon bundles and was already increased at 3dpi (Figure S6E). Closer analysis of the injury response with high‐power microscopy revealed augmented FASN and FABP7 localization in small membrane extensions inside the axon bundles during the phase of massive neurogenesis (14dpi, Figure 8E–H).

In addition, we found expression of MARCKS, a protein known to mediate membrane addition in oligodendrocyte maturation, on internal membrane compartments (Figure 8I). MARCKS is not only expressed by myelinating cells, but also is a marker for non‐myelinating Schwann cells (Yim et al. 2022). During injury, MARCKS was markedly upregulated in OEC membranes spanning the inside of the axon bundles (Figure 8K). We therefore speculate that membrane addition promotes OEC maturation and supports axon extension. Interestingly, the same processes were regulated in satellite glia cells of the dorsal root ganglion upon injury (Avraham et al. 2020).

## 4 Discussion

OECs exist in both the peripheral (lamina propria layer of the nasal mucosa) and central (olfactory nerve layer of the olfactory bulb) nervous system. OECs enwrap axon bundles in the lamina propria and support axonal growth to the glomerular targets in the olfactory bulb, and are particularly effective at recovering damaged neurons. OECs have been shown to have a reparative effect on the spinal cord (Khankan et al. 2016), facial nerves (Gu et al. 2019), sciatic nerves (Zhang et al. 2020), and brain injuries (Li et al. 2019), but the outcomes of clinical trials in OEC transplantation are variable, sometimes poor (Pearse et al. 2007). In order to improve OEC transplantation success, cellular and molecular biological characteristics of OECs need to be more thoroughly understood. Thus, the purpose of this study was to comprehensively characterize the response of OECs in the lamina propria to neuronal damage.

### 4.1 Steady‐State OECs Do Not Express NGFR

We describe here that the most commonly used marker to identify and purify OECs, NGFR, is expressed by lamina propria fibroblasts under homeostatic conditions. Moreover, *Ngfr* was not detected in olfactory bulb OECs by single‐cell sequencing (Tepe et al. 2018, #232) or in in situ hybridization profiles of the Allen Brain Atlas (Lein et al. 2007, #235). In response to injury, fibroblasts, horizontal basal cells, and OECs upregulate NGFR, which has potential pro‐healing capacity and modulates the healing processes in several tissue repair processes (Micera et al. 2007). *Ngfr* is also expressed in cultivated OECs from the rat olfactory bulb (Phelps et al. 2025), indicating that NGFR expression occurs in stress situations.

### 4.2 OEC Are Unique Glia Cells With Similarities to Satellite Glia Cells

OECs are non‐myelinating neural‐crest‐derived cells (Barraud et al. 2010) that ensheath axons of the olfactory sensory neurons. We compared the mucosal OEC transcriptome derived from published single cell RNA sequencing of the olfactory epithelium (Wang et al. 2022) to the transcriptomes of other glia cells (Zeisel et al. 2018). Molecularly, OECs express an unordinary combination of markers representative of oligodendrocytes (*Plp1*, *Sox10*), pericytes (*Vtn*), endothelial cells (*Cldn5*), neurons (*Npy*), Schwann cells (*Sox10*, *Plp22*), and astrocytes (*Gfap*). Interestingly, our comparison revealed that OECs from the lamina propria are equally similar to OECs from the olfactory bulb and to satellite glia cells, which envelop the neuronal soma in the dorsal root ganglion. Similarities to Schwann cells that insulate axons from sensory neurons are noticeably lower.

Moreover, lipid metabolism and PPARα signaling, the most important injury‐regulated processes in OECs in our study, were similarly regulated in satellite glia cells (Avraham et al. 2020). FASN, a key enzyme in the endogenous lipogenesis pathway (Chakravarthy et al. 2007; Menendez and Lupu 2007), participates in the regulation of regeneration‐associated genes, including *Atf3* and *Gap43*, in satellite glia cells (Avraham et al. 2020). FASN expression in OECs was higher in the phase of axon regeneration (14dpi) compared to later time points, correlating with expression levels of GAP43 (Senf et al. 2021). Moreover, OECs upregulate APOE, a PPARα target gene that is involved in lipid delivery for growth and regeneration of axons after nerve injury (Li et al. 2010; Vance et al. 2000, 1994), as well as genes involved in cholesterol metabolism and uptake, such as APOE, LRP1, and HMGCS1. Taken together, it is likely that regulation of lipid metabolism in OECs regulates axon regeneration through paracrine effects on neurons.

### 4.3 OECs Secrete Growth‐Permissive of Extracellular Matrix

One of the GO terms enriched upon injury in OECs was extracellular matrix. Brevican, which is involved in the control of neuronal plasticity, shows multiple associations with dementia, stroke, and movement functions (Liu et al. 2025). After CNS injury, production of chondroitin sulfate proteoglycans such as brevican by astrocytes increases sharply, leading to the inhibition of axon growth (Dyck and Karimi‐Abdolrezaee 2015). In the olfactory system, upregulation of extracellular matrix molecules does not seem to impair axon outgrowth, but the underlying mechanisms, possibly the molecular composition of the secreted matrix or the overall amount, remain to be studied.

### 4.4 OECs Do Not Proliferate in Response to Injury

A net increase in the number of OECs could be expected when the number of olfactory axons growing towards the olfactory bulb increases. Proliferation can be induced in cultured OECs by treatment with various compounds, such as TNFα (Lankford et al. 2017), sphingosine 1‐phosphate (Bao et al. 2020), natural polyphenol compound curcumin (Tello Velasquez et al. 2014), and alpha‐crystallin (Wang et al. 2012). Unexpectedly, we found proliferating macrophages, but OECs did barely respond to injury with proliferation, despite massive axonogenesis. Although unilateral bulbectomy led to OEC proliferation in neonatal mice (Chehrehasa et al. 2012), OECs did not divide at steady state or after intracranial section of the olfactory nerves in adult rats (Li et al. 2005) or in response to a lesion of the olfactory mucosa with ZnSO_4_ (Williams et al. 2004). Interestingly, also in the dorsal root ganglion, cell proliferation occurred in macrophages but not satellite glial cells after nerve injury (Avraham et al. 2020; Jager et al. 2020). Lack of proliferation may be linked to the absence of detectable SOX2 expression (Senf et al. 2021), since knockout of SOX2 inhibited proliferation of astrocytes and promoted the recovery of cortical tissue after postnatal brain injury (Liu et al. 2023). SOX2 is also required for proliferation of oligodendrocyte precursor cells during remyelination (Zhang et al. 2018). Lack of OEC proliferation might be compensated by horizontal basal cells, which can give rise to new OECs (Carter et al. 2004), but this remains to be investigated in future studies.

### 4.5 Perineural Fibroblasts Respond to Neuronal Injury

Perineural fibroblasts are extremely thin elongated cells that enwrap olfactory axon fascicles (Field et al. 2003; Li et al. 2005) and might function as mesenchymal stem cells (Delorme et al. 2010; Tomé et al. 2009). Fibroblasts form an anatomical barrier, possibly protecting the brain against pathogens that infect the nasal mucosa (Khan et al. 2022). As fibroblasts responded to nerve injury by upregulating NGFR and ICAM1, it appears possible that the repair capacity is influenced by their co‐existence with OECs. In the dorsal root ganglion, fibroblasts envelop neurons and attached satellite glial cells, and inhibit the communication of adjacent neurons (Zhang et al. 2022). Whether or not fibroblasts have stem cell properties, it is likely that they also play a role in olfactory epithelium maintenance and possibly need to be present in an optimal ratio with OECs.

### 4.6 OECs and Fibroblasts Express Proteins for Maintenance of Electrolyte Homeostasis

OECs express Claudin 5, the backbone of tight junctions forming the blood–brain barrier, with a key role in restricting the paracellular traffic of molecules and ions (Amasheh et al. 2005; Trevisani et al. 2025), which is not found in any other type of glial cell. Claudin 5 expression was maintained during nerve regeneration when new axons regrow through conduits formed and maintained in size and shape.

Another unique feature of OECs is the lack of aquaporin expression (Wang et al. 2022). Aquaporins are involved in diverse functions, such as regulation of extracellular space volume, potassium buffering, cerebrospinal fluid circulation, interstitial fluid resorption, waste clearance, and neuroinflammation. All types of astrocytes and satellite glial cells express *Aqp4*, while myelinating and Remak Schwann cells mostly express *Aqp1*. Perineural fibroblasts and fibroblasts close to the basal lamina express *Aqp1* and might therefore regulate water homeostasis. The lack of water channels might explain the stability of the OEC channel during regeneration.

Due to tight junctions in OECs, extracellular K^+^ inside of the axon bundle has to be regulated by OECs. We found that OECs express high levels of Na^+^K^+^‐ATPase in the membrane compartments inside of the axon bundles, which may be involved in K^+^ uptake from the extracellular space. Moreover, OECs express KCNJ10, a weak rectifying K^+^ channel, which allows movement of K^+^ depending on the transmembrane gradient, on inner membranes, and also on the outer limiting membrane of the axon bundles. Astrocytes and Müller cells of the retina buffer K^+^ concentrations predominantly through KCNJ10 to regulate the ionic and osmotic balance. KCNJ10 and Na^+^K^+^‐ATPase expression allows K^+^ uptake from the extracellular space after high neuronal activity or cell damage and release of K^+^ to the outside of the axon bundle. KCNJ10 localization shifted to the outer OEC membranes during regeneration, consistent with an increased need for K^+^ extrusion upon cellular damage.

OECs are connected among each other, but not with perineural fibroblasts, via gap junctions composed of Connexin 43. These gap junctions are lost during the regeneration of the olfactory neurons. Pharmacological blocking of gap junctions produces OEC hyperpolarization, which was explained by a shunting effect on KCNJ10 channels and a bigger impact of the KCNJ10 conductance on the resting potential (Rela et al. 2015). In this context, the loss of gap junctions in OEC during regeneration may improve K^+^ conductance and thereby facilitate K^+^ buffering.

## 5 Conclusions

Lamina propria OECs represent a promising treatment option for injuries of the nervous system, as the cells facilitate neural regeneration and can be obtained rather easily from biopsies of olfactory epithelium. Steady‐state analysis and analysis after olfactory nerve injury revealed that OECs and perineural fibroblasts react during regeneration. OECs showed marked similarities to satellite glial cells, but also unique properties that could be explored in future studies to improve the therapeutic effectiveness of OEC transplantation.

## Author Contributions

**K.S.** and **E.M.N.:** conception and design, data collection, analysis and interpretation, manuscript writing. **S.N.** and **M.W.:** data collection and analysis. All authors read and approved the final manuscript.

## Conflicts of Interest

The authors declare no conflicts of interest.

## Supporting information


**Figure S1:** Supplementary Figures.

**Table S1:** Antibodies.

## Data Availability

The data that supports the findings of this study are available in the Supporting Information of this article.
