# Supplementary material for: Unraveling the Role of Ensheathing Cells and Perineural Fibroblasts in Olfactory Neurogenesis
Source: Glia. 2025 Aug 7;73(12):2407–26. doi: 10.1002/glia.70076 (PMC12541900; doi:10.1002/glia.70076)
Supplement: Supplementary file 1 — Figure S1: Supplementary Figures. [file GLIA-73-2407-s001.pdf]

Supplementary Figures S1-S6, Senf et al.

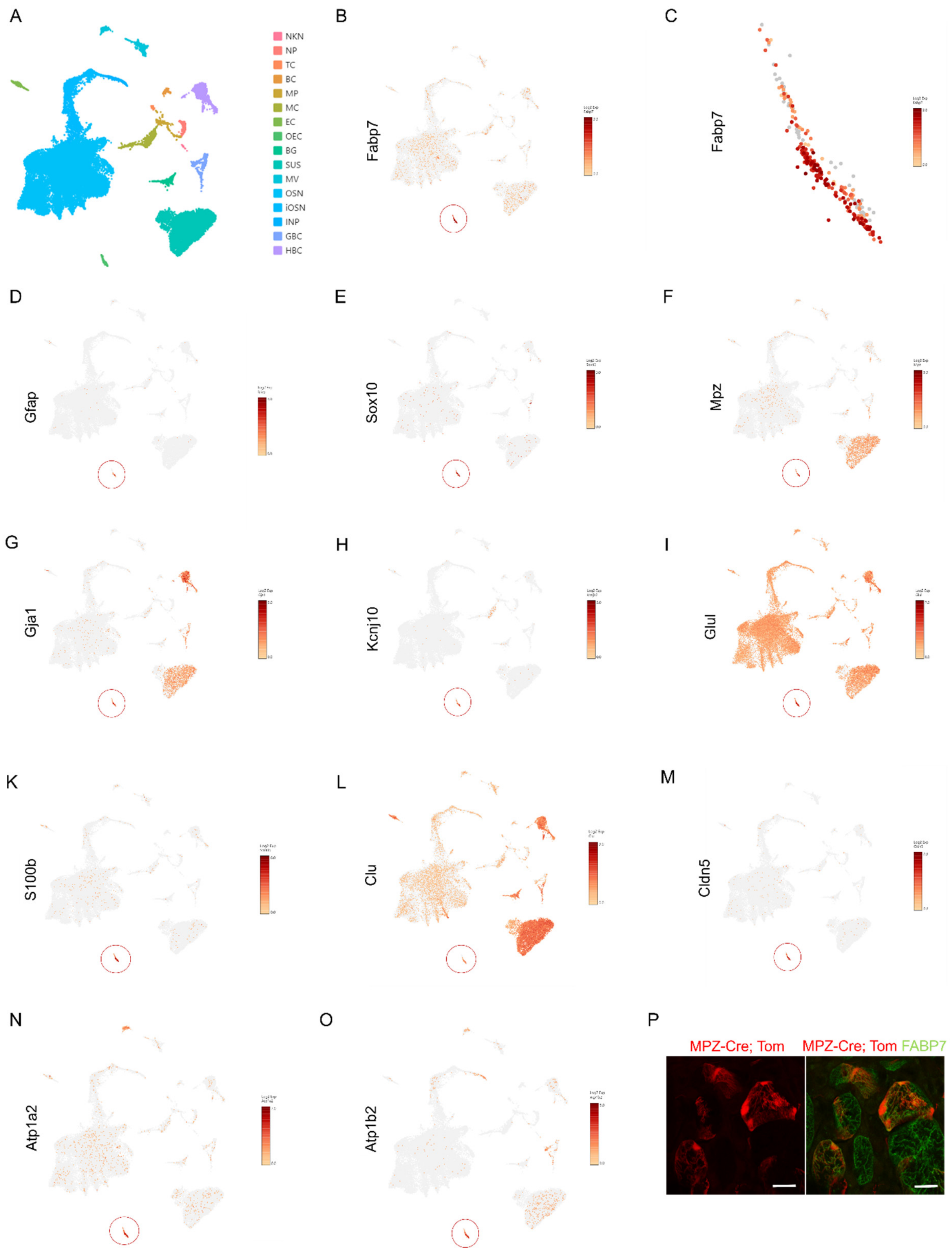

Figure S1

Expression distribution of genes analyzed in this study. (**A**) UMAP of cell type clusters of the olfactory mucosa from published single cell RNA sequencing data (Wang et al., 2022).

NKN unknown, NP neutrophils, TC T cell, BC B cell, MP macrophage, MC monocyte, EC ciliated respiratory cells, OEC olfactory ensheathing cell, BG Bowmann gland, SUS sustentacular cell, MV microvillar cell, OSN olfactory sensory neuron, iOSN immature olfactory sensory neuron, INP immediate neuronal precursor cell, GBC globose basal cell, HBC horizontal basal cell. UMAP plots of: (**B**) *Fabp7*, (**C**) expanded view on *Fabp7* expression in OEC cluster, showing different expression levels, (**D**) *Gfap*, (**E**) *Sox10*, (**F**) *Mpz*, (**G**) *Gja1*, (**H**) *Kcnj10*, (**I**) *Glul*, (**K**) *S100b*, (**L**) *Clu*, (**M**) *Cldn5,* (**N**) *Atp1a2,* (**O**) *Atp1b2.* OECs are located at the bottom of each image and are circled. (**P**) Representative immunofluorescence of 8W old Tg(*Mpz*-Cre);R26$^{CAG\text{-}LSL\text{-}tdT}$ mice, showing overlap of tomato and FABP7 as a marker for OECs. Scale bar 20µm.

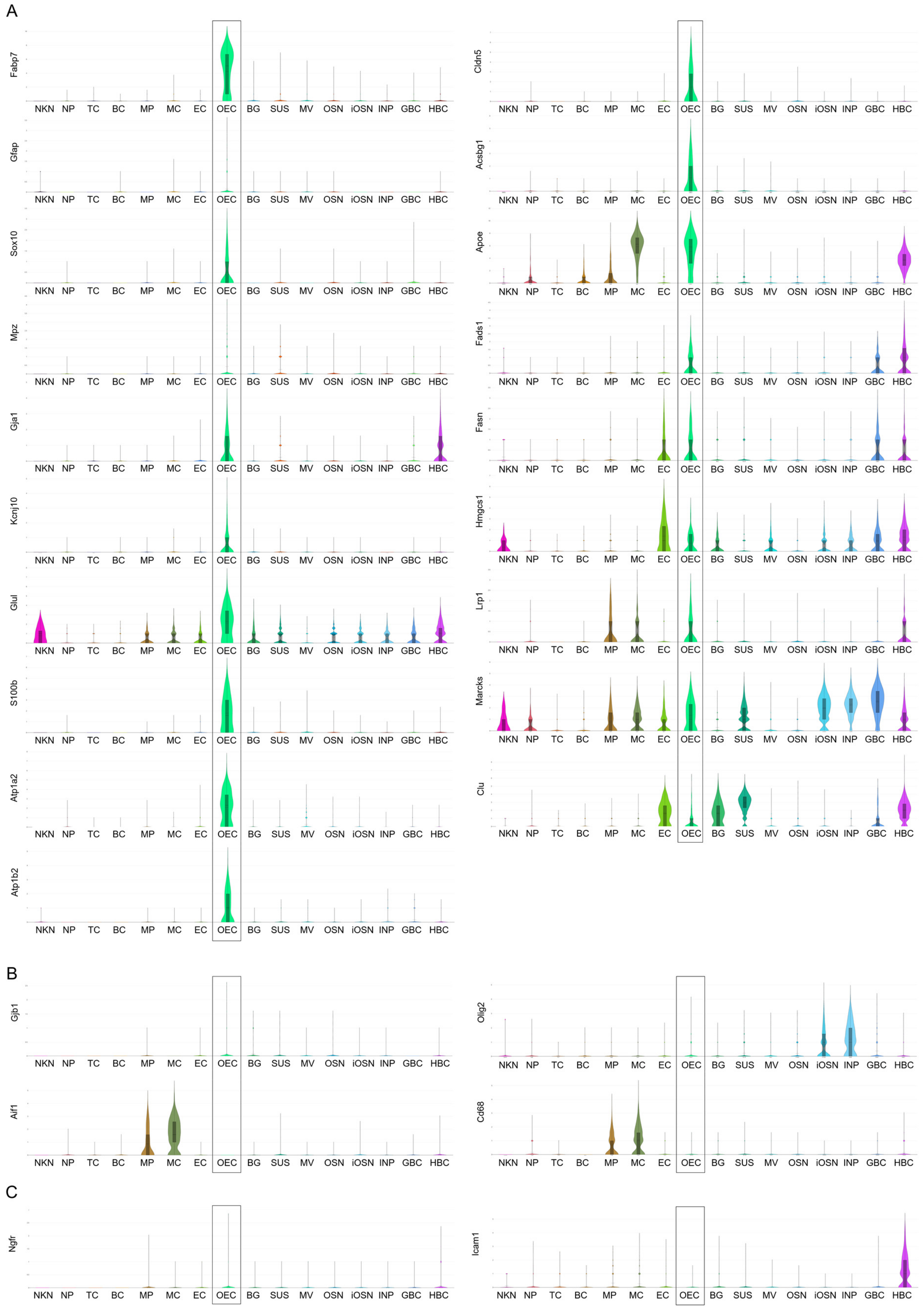
A
Fabp7
Gfap
Sox10
Mpz
Gja1
Kcnj10
Glul
S100b
Atp1a2
Atp1b2
Cldn5
Acsbg1
Apoe
Fads1
Fasn
Hmgcs1
Lrp1
Marcks
Clu
NKN NP TC BC MP MC EC OEC BG SUS MV OSN iOSN INP GBC HBC
B
Gjb1
Olig2
Aif1
Cd68
C
Ngfr
Icam1

Figure S2

Expression distribution of genes analyzed in this study. Shown are violin plots showing the expression distribution of genes analyzed in this study: (**A**) OEC expressed genes *Fabp7, Cldn5, Gfap, Ascbg1, Sox10, Apoe, Mpz, Fads1, Gja1, Fasn, Kcnj10, Hmgcs1, Glul, Lrp1, S100b, Marcks, Atp1a2, Clu, Atp1b2.* (**B**) Genes not expressed in OECs *Gjb1, Olig2, Aif1, Cd68.* (**C**) Expression of fibroblast marker genes in cells of the olfactory epithelium *Ngfr, Icam1.*

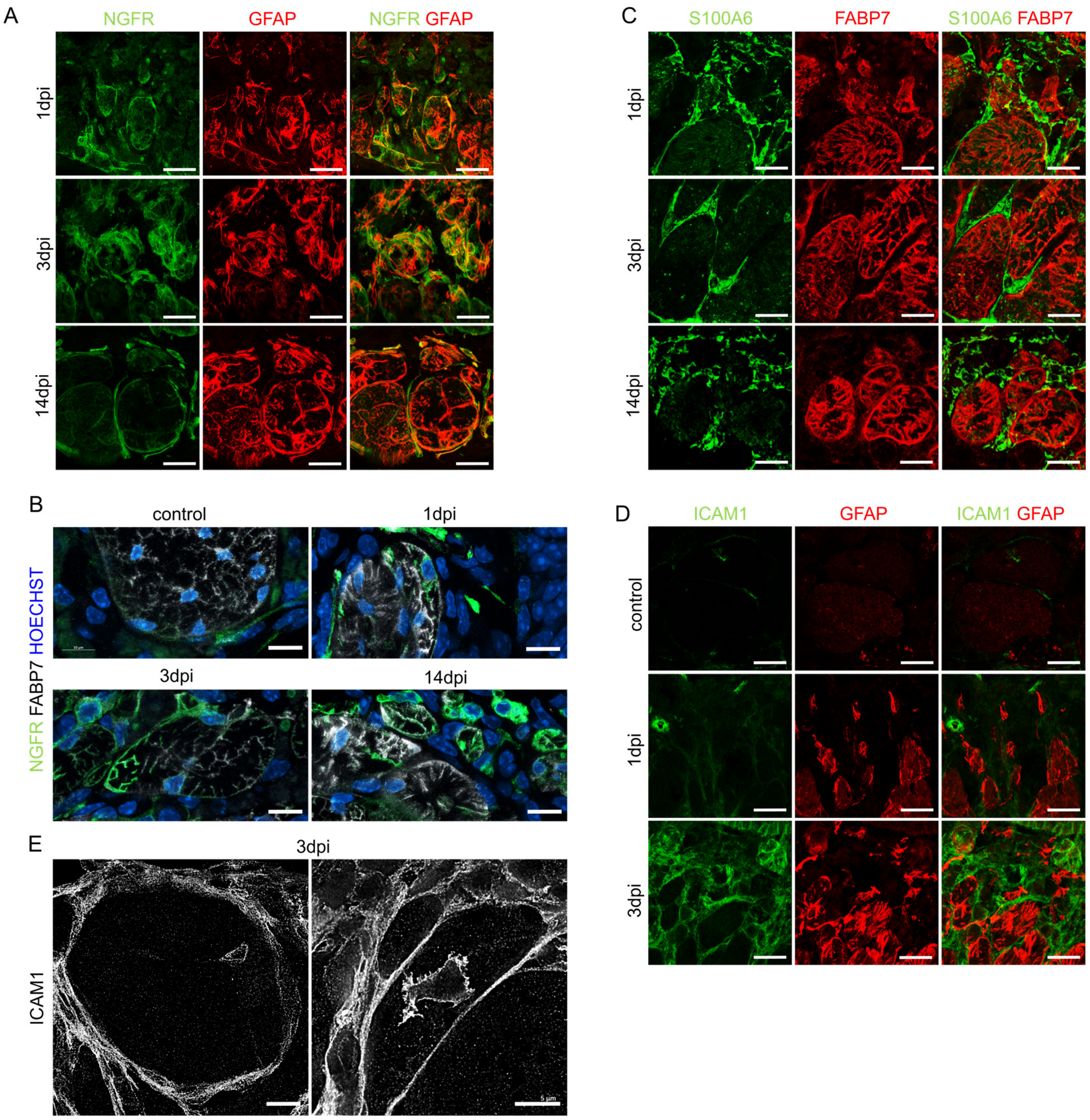

Figure S3

Different types of fibroblast are present in the lamina propria. (**A**) Representative immunofluorescence staining of NGFR (green) and GFAP (red) in the lamina propria of control WT mice, showing that OECs express NGFR after injury (1dpi, 3dpi, 14dpi). (**B**) NGFR (green) and FABP7 (white) co-localize after injury, NGFR is clearly localized on FABP7-positive membrane compartments extending through the axon bundle. (**C**) Fibroblasts and OEC remain different during regeneration, S100A6 (green) and FABP7 (red) never co-localized. (**D**) Immunofluorescence staining showing up-regulation of ICAM1 in fibroblasts during regeneration (1dpi, 3dpi). (**E**) Higher magnification of increased ICAM1 labeling surrounding the axon bundles. Scale bars (A), (C), (D), (E) 10µm, (B) 5µm.

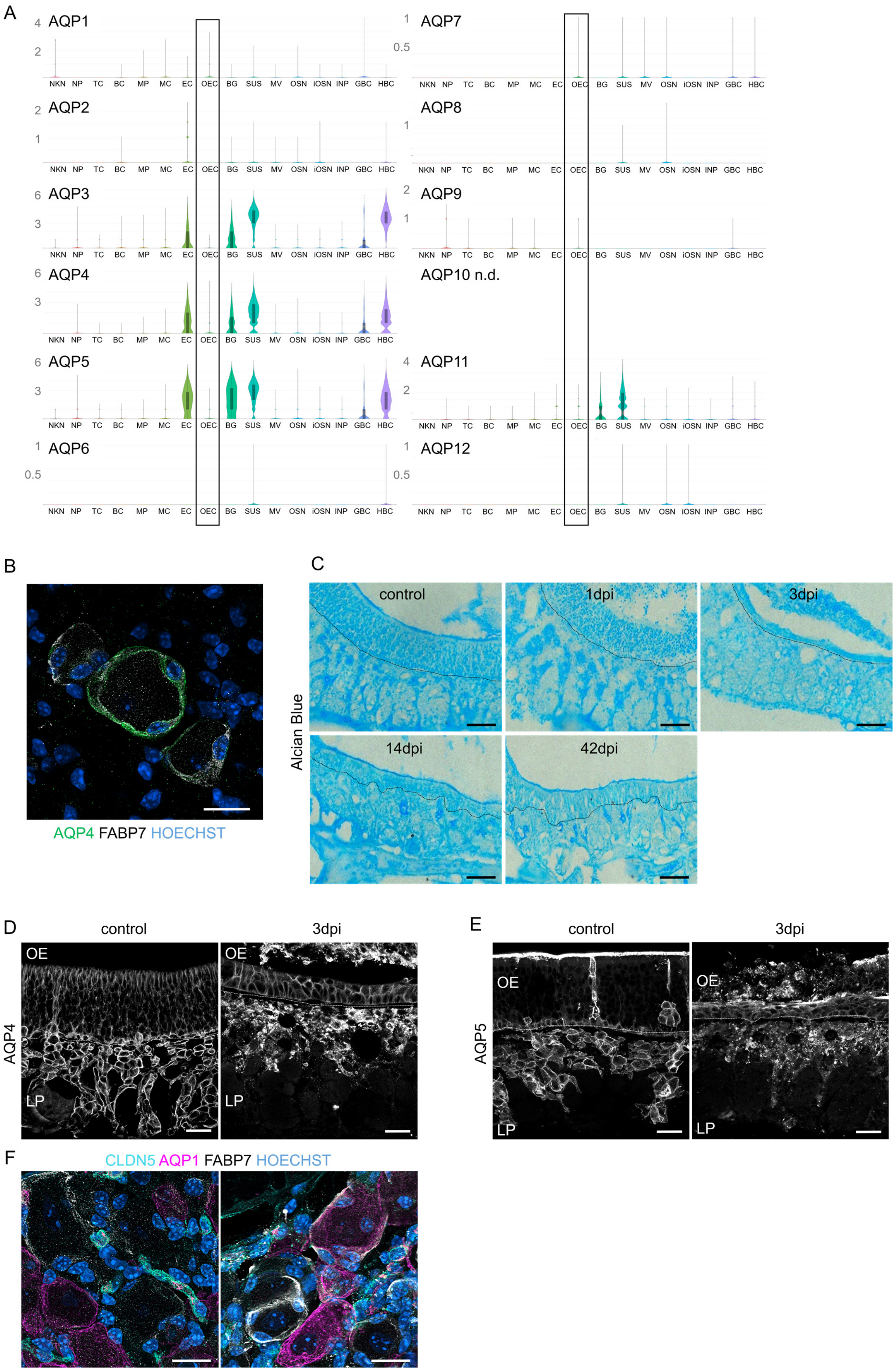
A
AQP1
AQP2
AQP3
AQP4
AQP5
AQP6
AQP7
AQP8
AQP9
AQP10 n.d.
AQP11
AQP12
NKN NP TC BC MP MC EC OEC BG SUS MV OSN iOSN INP GBC HBC
B
AQP4 FABP7 HOECHST
C
Alcian Blue
control
1dpi
3dpi
14dpi
42dpi
D
control
3dpi
AQP4
OE
LP
E
control
3dpi
AQP5
OE
LP
F
CLDN5 AQP1 FABP7 HOECHST

Figure S4

Electrolyte homeostasis in axon bundles. (**A**) Violin plots showing the expression of Aquaporin genes (*Aqp1-Aqp12*) in different cell types of the OE, derived from published data (Wang et al., 2022). OECs do not express Aquaporin genes. (**B**) Staining of AQP4 (green) and FABP7 (white) in satellite glia cells of the dorsal root ganglion. (**C**) Staining of used acidic polysaccharides in Bowmann Glands of the olfactory mucosa with Alcian blue showing absence of secreting gland cells after injury. (**D**) Staining of AQP4 under control conditions and at 3dpi. (**E**) Staining of AQP5 under control conditions and at 3dpi. (**F**) Staining of CLDN5 (cyan) in blood vessels of the dorsal root ganglion. Satellite glia cells are labeled in white, AQP1 (magenta) is expressed in fibroblasts and in some satellite glia. Dotted lines represent basal lamina. Scale bars 20µm.

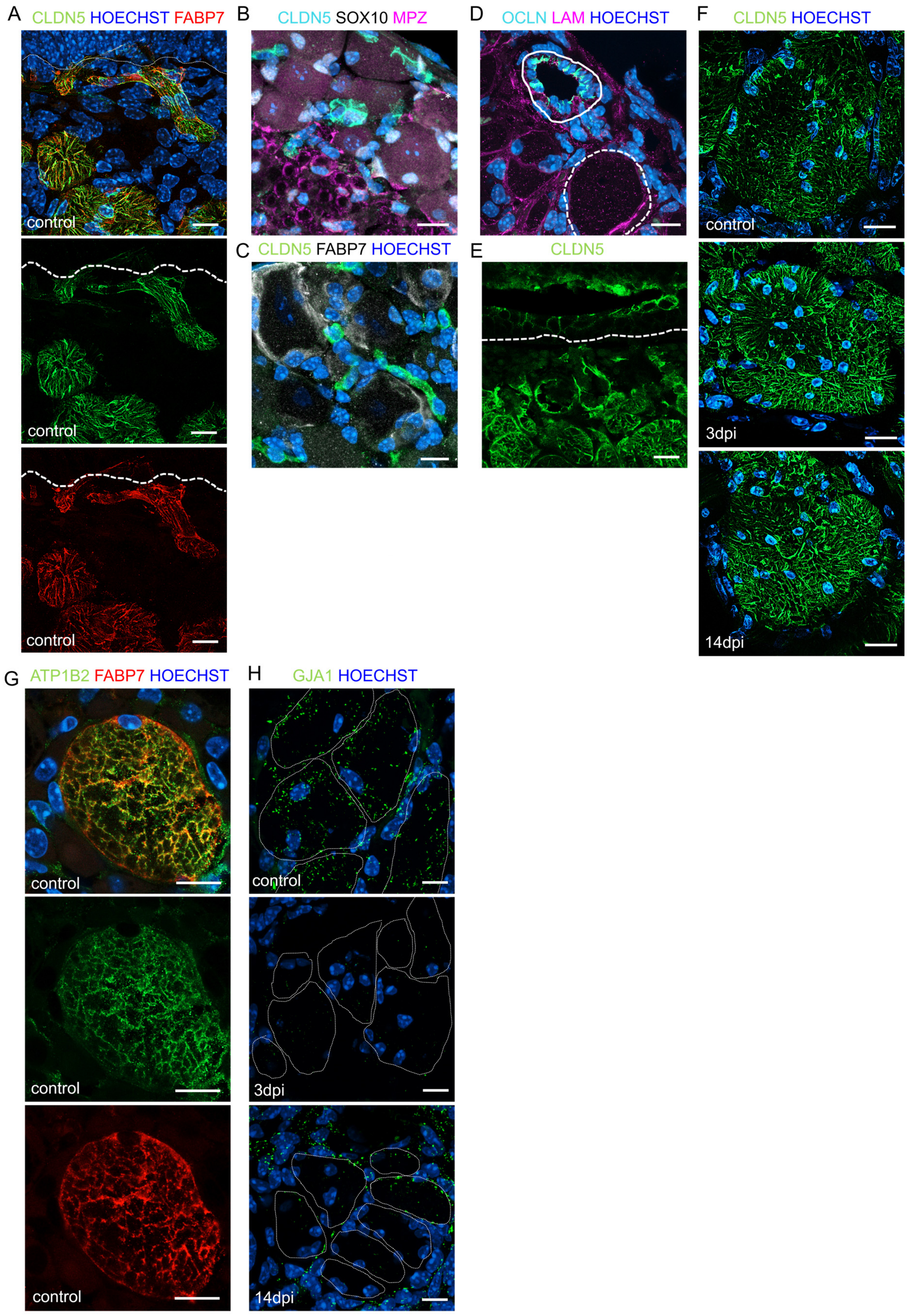

A
CLDN5 HOECHST FABP7
control
control
control
B
CLDN5 SOX10 MPZ
C
CLDN5 FABP7 HOECHST
D
OCLN LAM HOECHST
E
CLDN5
F
CLDN5 HOECHST
control
3dpi
14dpi
G
ATP1B2 FABP7 HOECHST
control
control
control
H
GJA1 HOECHST
control
3dpi
14dpi

Figure S5

OECs shield axon bundles. (**A**) CLDN5 (green) and FABP7 (red) co-localize in OECs. (**B**) CLDN5 (green) is expressed in capillaries of the dorsal root ganglion. Satellite glia cells (nuclei labeled with SOX10, white) surrounding neuronal cell bodies and Schwann cells (labeled with MPZ, red) do not express CLDN5.(**C**) CLDN5 (green) labels blood vessels FABP7-positive satellite glia cells (white) are not labeled in the dorsal root ganglion. (**D**) Labeling of Occludin (OCLN) in blood vessels (solid line) of the lamina propria, OECs/axon bundles are not labeled (dashed line). (**E**) Olfactory epithelium at 3dpi. The upper row of activated horizontal basal cells express CLDN5 (green). (**F**) OECs in large axon bundles express CLDN5 (green). CLDN5 staining was increased after injury (3dpi, 14dpi). (**G**) Staining of ATP1B2 (red) in axon bundles showing localization on membranes extending through the axon bundle, but also on the part of the OEC membrane lining the outer border of the axon bundle, ATP1B2 co-localized with FABP7 (green). Nuclei are stained with Hoechst (blue) (**H**) GJA1 staining (green) in axon bundles disappears after injury and does not recover within 2 weeks. Nuclei are stained with Hoechst (blue). Dotted lines represent basal lamina or encircle axon bundles. Scale bars 10µm.

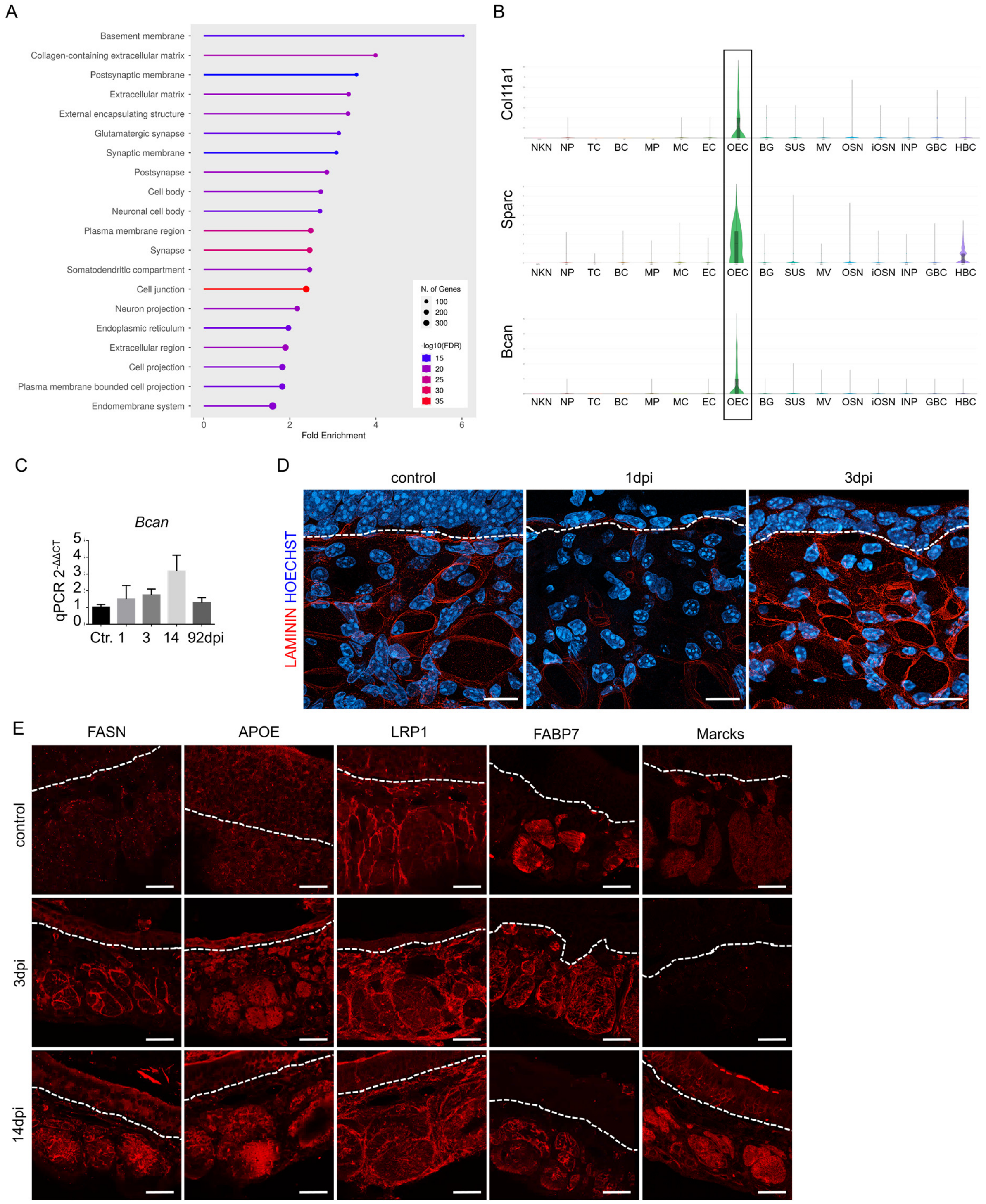

Figure S6

Laminin dynamics in the lamina propria (**A**) GO analysis of the regulated OEC genes for cellular component revealed overrepresentation of "basement membrane". (**B**) Violin plots showing the expression of extracellular matrix genes (*Col11a1, Sparc, Bcan*) in different cell types of the OE, derived from published data (Wang et al., 2022). (**C**) Quantitative PCR of *Bcan* (relative to *Gpdh*). Student's t test, differences are not significant. (**D**) Staining of Laminin A under control conditions, at 1dpi and at 3dpi. At 1dpi, laminin (red) was strongly reduced, but

was already recovered at 3dpi. Nuclei are stained with Hoechst (blue). (**E**) Staining of lipid metabolism proteins in the lamina propria during regeneration: FASN, APOE, LRP1, FABP7 and MARCKS. Dotted lines represent the basal lamina. Scale bars 20μm.

References

Wang, I. H., Murray, E., Andrews, G., Jiang, H. C., Park, S. J., Donnard, E., . . . Greer, P. L. (2022). Spatial transcriptomic reconstruction of the mouse olfactory glomerular map suggests principles of odor processing. *Nat Neurosci, 25*(4), 484-492. doi:10.1038/s41593-022-01030-8
