# Supplementary material for: Unraveling the Role of Ensheathing Cells and Perineural Fibroblasts in Olfactory Neurogenesis
Source: Glia. 2025 Aug 7;73(12):2407–26. doi: 10.1002/glia.70076 (PMC12541900; doi:10.1002/glia.70076)
Supplement: Supplementary file 2 — Table S1: Antibodies. [file GLIA-73-2407-s002.docx]

| **PRIMARY ANTIBODIES** | **HOST SPECIES** | **COMPANY, ORDER NUMBER** | **DILUTION** |
| --- | --- | --- | --- |
| Lrp1 | rabbit | Abcam, ab92544  monoclonal | 1:100 |
| Plat (t-PA) | rabbit | Proteintech 10147-1-AP | 1:100 |
| Brevican | rabbit | Proteintech 19017-1-AP | 1:500 |
| Brevican | rabbit | BD Bioscience 610894 | 1:50 |
| Apoe | goat | Merck, AB947 | 1:100 |
| FASN | rabbit | Abcam, ab128870 | 1:100 |
| KCNJ10 | guinea pig | Alomone APC-035-GP | 1:100 |
| Aqp1 | rabbit | Alomone AQP-001 | 1:200 |
| Aqp1 | rabbit | Proteintech 20333-1-AP | 1:200 |
| Fabp7 | goat | R&D Systems AF3166 | 1:200 |
| Gng8 | rabbit | Antibodies Online ABIN7443331 | 1:50 |
| Dcx | guinea pig | Synaptic Systems 326 004 | 1:200 |
| Cxcr4 | rat | Affymetrix 14-9991-82 | 1:200 |
| Gap43 | mouse | Millipore MAB347 | 1:100 |
| Omp | goat | Wako 544-10001 | 1:300 |
| Gfap | Guinea pig | Synaptic Systems 173 003 | 1:200 |
| Sox10 | Goat | R&D Systems AF2864 | 1:100 |
| Mpz | Rabbit | Genetex 134079 | 1:100 |
| Gjb1 (Cx32) | Rabbit | Proteintech 10450-1-AP | 1:50 |
| Gja1 (Cx43) | mouse | Thermo Fisher 35-5000 | 1:100 |
| Olig2 | Goat | R&D Systems AF-2418-SP | 1:100 |
| Aif1 (Iba1) | Rabbit | Genetex GTX101495 | 1:100 |
| Cd68 | Rabbit | Genetex GTX134008 | 1:50 |
| Aqp4 | Rabbit | Genetex GTX133151 | 1:100 |
| Glutamine Synthetase (GS-6) | Mouse | Sigma 389M-18 | 1:25 |
| S100b | Rabbit | Genetex GTX129573 | 1:100 |
| 4-Hydroxynonenal | Mouse | R&D Systems MAB3249 | 1:50 |
| Lamp1 | Rat | R&D Systems AF2864 | 1:100 |
| Clu (Clusterin) | Rabbit | Genetex GTX31733 | 1:100 |
| Caspase-3 | Rabbit | Cell Signaling 9664S | 1:100 |
| Krt5 | Rabbit | BioLegend 905501 | 1:200 |
| Mcm2 | Mouse | BD Biosciences 610700 | 1:50 |
| Ki67 | Mouse | BD Biosciences 550609 | 1:50 |
| Arl13b | Mouse | Neuromab n295b/66  75-287 | 1:100 |
| Tubb3 | Rabbit | Biolegend | 1:100 |
| Ngfr (P75) | Rabbit | Abcam Ab52987 | 1:50 |
| S100a6 | Rabbit | Sigma HPA007575 | 1:100 |
| Icam1 | Goat | R&D Systems AF796 | 1:300 |
| Scd1 (syndecan) | Rat | BD Pharmingen 553712 | 1:100 |
| Cldn5 | Mouse | Invitrogen 35-2500 | 1:200 |
| Atp1a2 | Rabbit | Proteintech | 1:200 |
| Atp1b2 | Rabbit | Proteintech 22338-1-AP | 1:50 |
| Aqp5 | Rabbit | Millipore 178615 | 1:200 |
| Ocln (Occludin) | Mouse | Thermo Fisher 331500 | 1:100 |
| Lam (Laminin) | Rabbit | Millipore L9393 | 1:100 |
| tdTomato | Goat | Sicgen AB8181 | 1:200 |
| Marcks | Rabbit | Proteintech 20661-I-AP | 1:50 |
|  |  |  |  |
| **Secondary ANTIBODIES** | **HOST SPECIES** | **COMPANY, ORDER NUMBER** | **DILUTION** |
| Donkey anti goat Alexa Fluor® 488 | Thermo Fisher Scientific | Cat# A-11055 | 1:500 |
| Donkey anti rabbit Alexa Fluor® 488 | Thermo Fisher Scientific | Cat# A-21206 | 1:500 |
| Donkey anti rat Alexa Fluor® 488 | Thermo Fisher Scientific | Cat# A-21208 | 1:500 |
| Donkey anti mouse Alexa Fluor® 488 | Thermo Fisher Scientific | Cat# A21202 | 1:500 |
| Donkey anti guinea pig Alexa Fluor® 488 | Dianova | Cat# 706-545-148 | 1:500 |
| Donkey anti goat Alexa Fluor® 568 | Thermo Fisher Scientific | Cat# A-11057 | 1:500 |
| Donkey anti mouse Alexa Fluor® 568 | Thermo Fisher Scientific | Cat# A-10037 | 1:500 |
| Donkey anti rabbit Alexa Fluor® 568 | Thermo Fisher Scientific | Cat# A-10042 | 1:500 |
| Donkey anti rat Rhodamin RedX | Thermo Fisher Scientific | Cat# 712-295-153 | 1:500 |
| Donkey anti guinea pig Alexa Fluor® 568 | Dianova | Cat# 706-295-148 | 1:500 |
| Donkey anti rabbit Alexa Fluor® 647 | Thermo Fisher Scientific | Cat# A-31573 | 1:500 |
| Donkey anti rat Alexa Fluor® 647 | Dianova | Cat# 712-605-153 | 1:500 |
| Donkey anti goat Alexa Fluor® 647 | Thermo Fisher Scientific | Cat# A32849 | 1:500 |
| Donkey anti guinea pig Alexa Fluor® 647 | Dianova | Cat# 706-605-148 | 1:500 |
| Donkey anti mouse Alexa Fluor® 647 | Thermo Fisher Scientific | Cat# A-31571 | 1:500 |
| Hoechst3342 | Thermo Fisher Scientific | Cat# 62249 | 1:500 |

Table S1: Antibodies.
